# Comparative transcriptome analysis of a lowly virulent strain of *Erwinia amylovora* in shoots of two apple cultivars – susceptible and resistant to fire blight

**DOI:** 10.1186/s12864-017-4251-z

**Published:** 2017-11-13

**Authors:** Joanna Puławska, Monika Kałużna, Wojciech Warabieda, Artur Mikiciński

**Affiliations:** Research Institute of Horticulture, ul. Konstytucji 3 Maja 1/3, 96-100 Skierniewice, Poland

**Keywords:** Fire blight, RNA-seq, Virulence

## Abstract

**Background:**

*Erwinia amylovora* is generally considered to be a homogeneous species in terms of phenotypic and genetic features. However, strains show variation in their virulence, particularly on hosts with different susceptibility to fire blight. We applied the RNA-seq technique to elucidate transcriptome-level changes of the lowly virulent *E. amylovora* 650 strain during infection of shoots of susceptible (Idared) and resistant (Free Redstar) apple cultivars.

**Results:**

The highest number of differentially expressed *E. amylovora* genes between the two apple genotypes was observed at 24 h after inoculation. Six days after inoculation, only a few bacterial genes were differentially expressed in the susceptible and resistant apple cultivars. The analysis of differentially expressed gene functions showed that generally, higher expression of genes related to stress response and defence against toxic compounds was observed in Free Redstar. Also in this cultivar, higher expression of flagellar genes (FlaI), which are recognized as PAMP (pathogen-associated molecular pattern) by the innate immune systems of plants, was noted. Additionally, several genes that have not yet been proven to play a role in the pathogenic abilities of *E. amylovora* were found to be differentially expressed in the two apple cultivars.

**Conclusions:**

This RNA-seq analysis generated a novel dataset describing the transcriptional response of the lowly virulent strain of *E. amylovora* in susceptible and resistant apple cultivar. Most genes were regulated in the same way in both apple cultivars, but there were also some cultivar-specific responses suggesting that the environment in Free Redstar is more stressful for bacteria what can be the reason of their inability to infect of this cultivar. Among genes with the highest fold change in expression between experimental combinations or with the highest transcript abundance, there are many genes without ascribed functions, which have never been tested for their role in pathogenicity. Overall, this study provides the first transcriptional profile by RNA-seq of *E. amylovora* during infection of a host plant and insights into the transcriptional response of this pathogen in the environments of susceptible and resistant apple plants.

**Electronic supplementary material:**

The online version of this article (10.1186/s12864-017-4251-z) contains supplementary material, which is available to authorized users.

## Background

*Erwinia amylovora* is the causal agent of fire blight, occurring on over 130 plant species belonging to 40 genera, mainly from the family *Rosaceae* [1]. It is a serious bacterial pathogen, causing severe loses in production of apples and pears worldwide. The symptoms of fire blight can be observed on all above-ground parts of the plant. The most common are wilt and death of flowers; dieback of shoots, twigs, leaves, and fruits; and cankers of branches and the trunk, which can cause the dieback of the whole plant.

The pathogenic abilities of *E. amylovora* are determined by several factors. Based on present knowledge, the most important are the type III secretion system (T3SS) and biosynthesis of exopolysaccharides (EPS) amylovoran and levan. *E. amylovora,* as for many other pathogenic bacteria, uses T3SS to deliver effector proteins (T3E) into the cytosol of host plants. In the host cell, T3Es exert a number of effects that help the pathogen to survive and to escape immune response [2]. Exopolysaccharides play a role in bypassing the plant defence system, in blocking the vascular system of the plant and in protecting the bacteria against water and nutrient loss during dry conditions and the toxic effect of reactive oxygen species (ROS) [3, 4]. Additionally, they are crucial in the formation of biofilm, which is essential for attachment to several surfaces and for pathogenicity of bacteria [5].

To establish a pathogenic relationship with a host plant, *E. amylovora* uses complex regulatory systems that sense environmental signals and induce virulence genes. These systems include two component signal transduction systems (TCSTs), regulating amylovoran biosynthesis and swarming motility [6, 7], c-di-GMP, which positively regulates the secretion of the main exopolysaccharide in *E. amylovora*, amylovoran, leading to increased biofilm formation and quorum sensing [8, 9], the bacterial alarmone ppGpp, crucial for T3SS regulation [10], and small RNAs (sRNAs) [11]. For successful infection other important factors are: i/ motility [12], ii/ biofilm formation [5], iii/ adhesion [13], iv/ stress responses including efficient expulsion of a wide range of compounds toxic to bacteria [14, 15], v/ resistance towards host plant toxins, such as phytoalexins [16], vi/ adaptation to the environmental niche via catabolism of available carbohydrates, such as sucrose [17] and sorbitol [18], vii/ production of siderophores for iron acquisition [19], viii/ the production of metalloproteases, which are important for tissue colonization [20].

The *E. amylovora* strains collected worldwide have been found to be very similar in terms of phenotypic and genetic features, as reviewed by Puławska and Sobiczewski [21]; however, they are quite different in their levels of virulence [22]. The difference in virulence of particular strains was observed mostly on hosts with different susceptibility to fire blight, e.g., different apple cultivars. Some strains are able to infect only susceptible cultivars, while others can also infect cultivars that are found to be resistant to fire blight [23, 24]*.* One qualitative difference between strains responsible for overcoming resistance to fire blight of *Malus* × *robusta* 5 is that the single nucleotide polymorphism (SNP) resulting in an exchange of cysteine to serine was detected in type 3 effector (T3E) *avrRpt2*_*EA*_ [25]. The difference in virulence between *E. amylovora* strains can have also quantitative background e.g., the amount of amylovoran produced and the expression of genes crucial for pathogenicity [23, 26]*.* However, no complex studies revealing the differences at the transcriptome level have been performed to date.

The infection of apple plants by *E. amylovora* elicits several mechanisms related to plant defence. These plant defence responses include various molecular, physiological and cellular processes, activation of expression of multiple genes, and accumulation of secondary metabolites. These processes involve a hypersensitivity response, which leads to building systemic acquired resistance, an oxidative burst, cutin formation and callose deposition [27, 28].

The available data show that resistance to fire blight in apples is based on several mechanisms involving various pathways. Several QTLs (Quantitative Trait Loci) related to resistance to fire blight in different apple genetic backgrounds and in response to different *E. amylovora* strains have been found [reviewed in [29]. Comparative studies of the reaction of sensitive and resistant apple cultivars to *E. amylovora* infection have revealed higher expression of a gene encoding vacuolar processing enzyme (VPE) - a caspase-like protease active during programmed cell death [30], BAX inhibitor and HIR proteins involved in hypersensitivity reactions and controlled cell death, and proteins involved in signal transduction, especially serine/threonine kinase and β-1,3-glucanase (PR-2 protein) [31]. Milcevičová et al. [32] indicated that the resistant plants might represent a less favourable environment for bacterial growth and have higher levels of some defence-related compounds, such as salicylic acid, or increased activities of these compounds, such as the PAL enzyme. Additionally, the levels of phenolic compounds, which are potential inhibitors of *E. amylovora,* are higher in resistant plants [33].

The aim of our study was to decipher differences in the response of a lowly virulent *E. amylovora* strain to infection of susceptible and resistant apple cultivars at the transcriptome level. For this purpose, we applied an RNA-seq technique to see the global changes in gene expression of *E. amylovora* while interacting with two apple cultivars at two time points after inoculation of shoots. We believed to find differences resulting in the inability to infect the resistant apple cultivar. Additionally, we compared transcriptomes of *E. amylovora* growing on a microbiological medium and *in planta* to elucidate transcriptional changes in bacterial cells induced by the host plant environment*.* Until now, no detailed studies on the mechanism of action of *E. amylovora* on hosts with different susceptibility levels have been carried out. This is also the first study to apply an RNA-seq technique for analysis of *E. amylovora* gene expression*.*

## Results

### *E. amylovora* virulence test

**Fig. 1:**
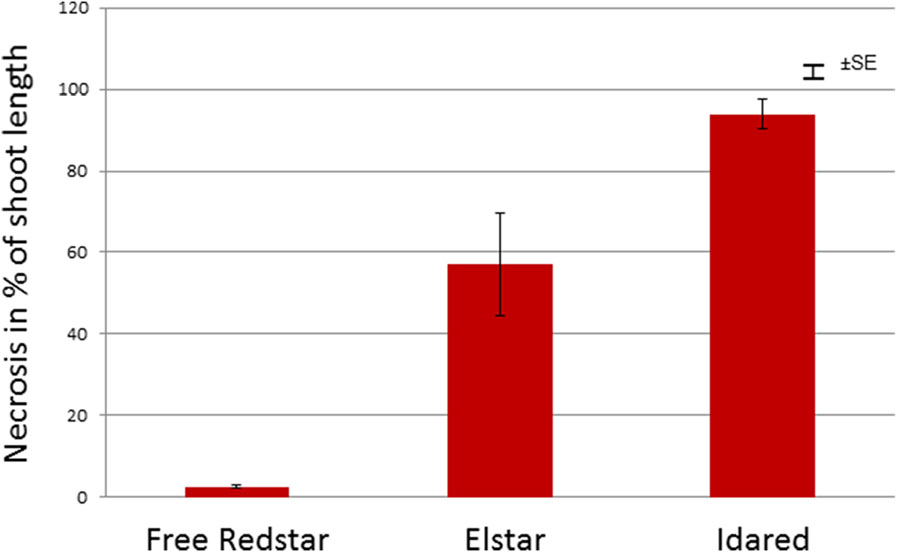
Mean virulence rating of *E. amylovora* 650 strain used for inoculation of actively growing shoots of three apple cultivars of different susceptibility. Virulence was measured 6 weeks post-inoculation and it is expressed as a percent of the length of a shoot exhibiting necrosis divided by the entire length of shoot. Mean virulence ratings were separated with Tukey’s test at a significance level of *P* = 0.05. The vertical bars represent standard error

The analysis of virulence of *E. amylovora* strain 650 revealed differences in its ability to infect the different apple genotypes. The most intensive disease symptoms – 94.1% – were observed on the cv. Idared, known to be susceptible to fire blight. On the middle susceptible cv. Elstar the virulence was 57.2%, while on the cv. Free Redstar, known to be resistant to fire blight, the virulence of strain 650 was estimated to be 2.6% (Fig. 1). In the view of this results and earlier studies [34], strain 650 could be classified as lowly virulent strain.

### Overview of RNA-seq results

**Fig. 2:**
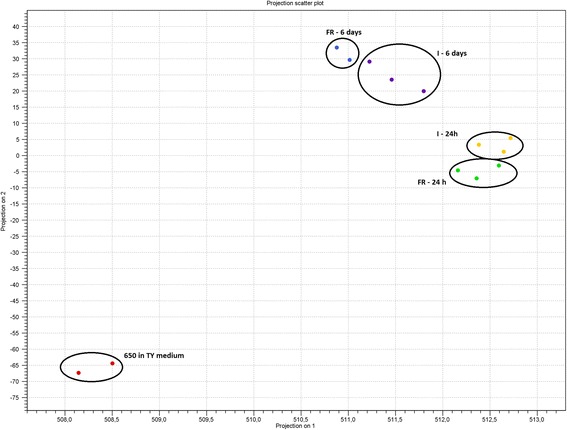
The Principal Component Analysis (PCA) of the log_2_ - transformed normalized expression values highlighted the variability between the samples. FR – Free Redstar, I – Idared, 24 h – 24 h after inoculation, 6 days – 6 days after inoculation

For each biological replicate, the library was constructed and sequenced on the MiSeq (Illumina). For each sample, from 4,628,510 to 17,099,456 reads were obtained, and 1,320,224 to 10,215,328 reads were mapped to genes of *E. amylovora* CFBP 1430 genome (Additional file 1: Table S1). Mapping to rRNA operons showed that one replicate, FR-650-6d-3, possessed over 72.95% of reads complementary to rRNA (Additional file 1: Table S1), although analysis of this sample on a 2100 Bioanalyzer (Agilent) before sequencing did not show any traces of rRNA. This sample was eliminated from further analysis. The rest of the biological replicates showed a very high level of correlation (*r* ≥ 0.99). The Principal Component Analysis (PCA) of the log2-transformed normalized expression values highlighted the variability among the samples and revealed the influence of different environmental conditions on bacterial gene expression (Fig. 2).

**Fig. 3:**
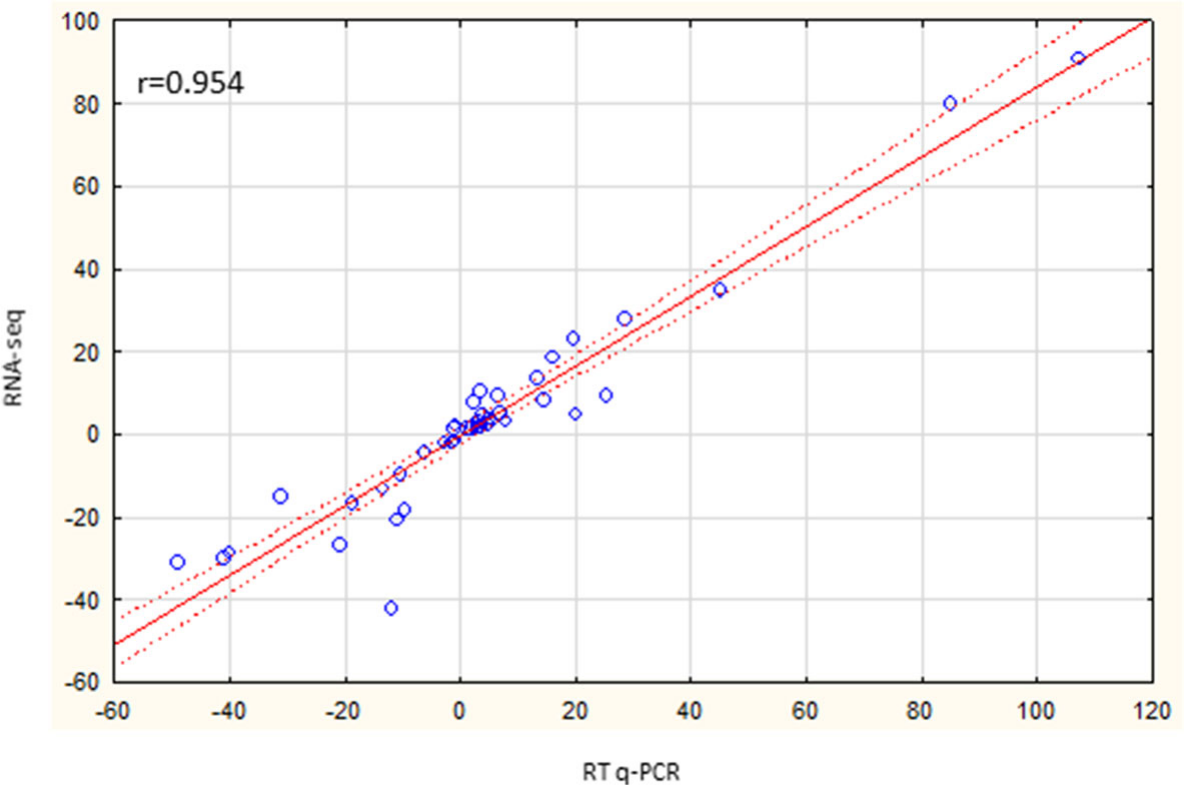
Validation of RNA-seq data using RT-qPCR. Fold changes of gene expression detected by RNA-seq were plotted against the data of qPCR. The reference line indicates the linear relationship between the results of RNA-seq and qPCR

The accuracy of the RNA-seq data was verified using reverse transcription quantitative real-time polymerase chain reaction, RT-qPCR. Fold changes in expression values under different experimental conditions obtained with these two techniques were plotted on a scatter graph, with fold change values obtained from RT-qPCR on the X-axis and those obtained from RNA-seq on the Y-axis (Fig. 3). A high value for the Pearson correlation coefficient (*r* = 0.954; *p* < 0.001; R^2^ = 0.909) indicated a positive correlation between the two variables.

### Expression of *E. amylovora* 650 genes *in planta*

**Fig. 4:**
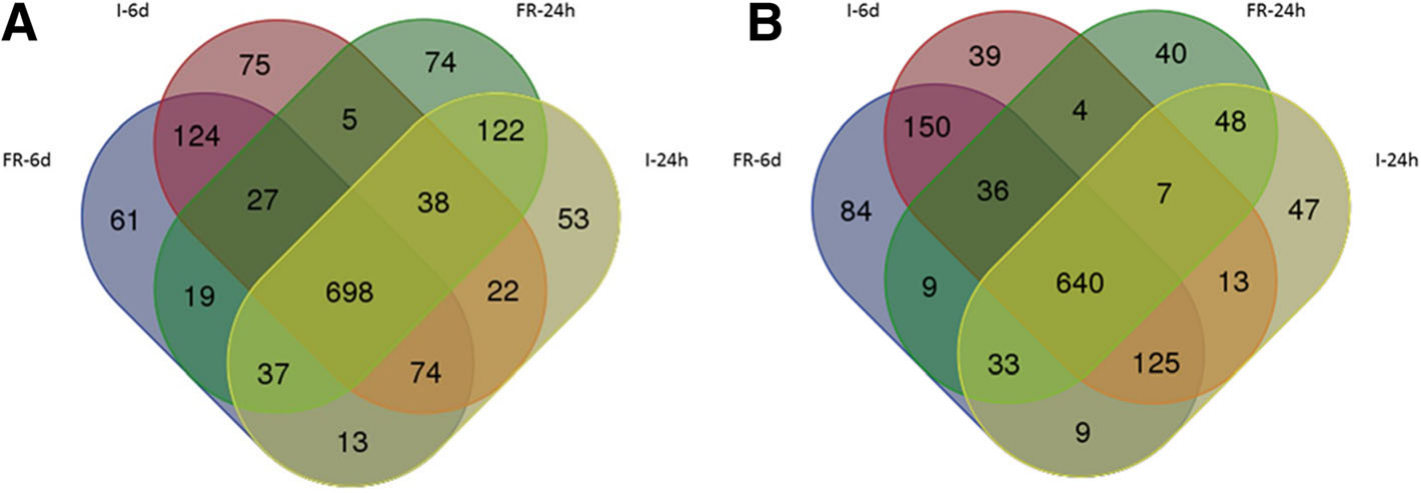
Venn diagram representing number of *E. amylovora* genes **a**/ up- and **b**/ down-regulated *in planta* comparing to expression of genes of pure bacterial culture grown overnight in TY medium. I – Idared; FR – Free Redstar; 24 h – sample collected 24 h after inoculation; 6d - sample collected 6 days after inoculation

**Fig. 5:**
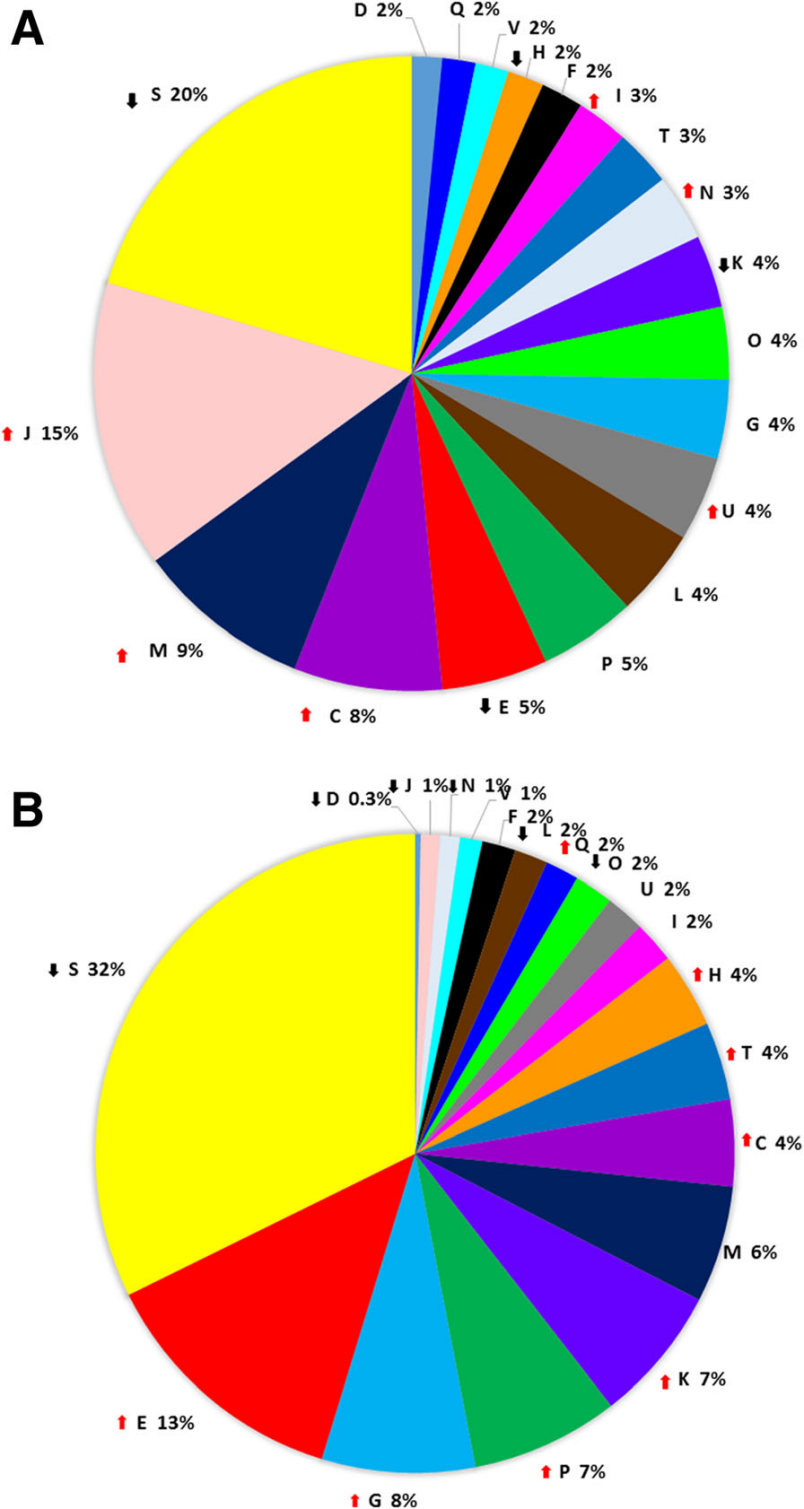
Relative abundance of CDSs assigned to each eggNOG functional categories for commonly a/ 640 down and b/ 698 up-regulated *E. amylovora* genes *in planta*. The eggNOG functional categories are as follows: C, energy production and conversion; D, cell cycle control, cell division and chromosome partitioning; E, amino acid transport and metabolism; F, nucleotide transport and metabolism; G, carbohydrate transport and metabolism; H, coenzyme transport and metabolism; I, lipid transport and metabolism; J, translation; K, transcription; L, replication; M, cell wall/membrane/envelope biogenesis; N, cell motility; O, posttranslational modification, protein turnover, chaperones; P, inorganic ion transport and metabolism; Q, secondary metabolites biosynthesis, transport and catabolism; S, function unknown; T, signal transduction mechanisms; U, intracellular trafficking and secretion; V, defence mechanisms. ↑- over-represented; ↓ - under-represented COG categories calculated based on hypergeometric distribution at FDR < 0.05

Over 50% of the *E. amylovora* 650 genes were differentially expressed *in planta* both at 24 h and 6 days after inoculation of two apple cultivars compared to the transcriptome of bacteria in pure culture in liquid TY medium. A total of 640 down-regulated genes and a set of another 698 up-regulated genes were found for both apple cultivars at the two time points after inoculation (Table 1, Fig. 4, Fig. 5).

**Table 1:** Number of differentially expressed genes between experimental combinations

	650-bact	I-24 h	I-6d	FR-24 h	FR-6d
650-bact	–	1057 ↑ 922 ↓	1063 ↑ 1014 ↓	1020 ↑ 817↓	1053 ↑ 1086 ↓
I-24 h	922 ↑ 1057 ↓	–	519 ↑ 494 ↓	142 ↑ 150 ↓	na
I-6d	1014 ↑ 1063 ↓	494 ↑ 519 ↓	–	na	6 ↑ 4 ↓
FR-24 h	817 ↑ 1020 ↓	150 ↑ 142 ↓	na	–	572 ↑ 510 ↓
FR-6d	1086 ↑ 1053 ↓	na	4 ↑ 6 ↓	510 ↑ 572 ↓	–

650 – *E. amylovora* strain used in the study; bact – RNA isolated from pure bacterial culture; I – Idared; FR – Free Redstar; 24 h – sample collected 24 h after inoculation; 6d - sample collected 6 days after inoculation; na – not analysed

↑ - up-regulated and ↓- down-regulated genes of the samples listed in the first row in relation to the samples listed in the first column

The 640 down- and 698 up-regulated genes were classified into the same 19 eggNOG/COG categories. In the case of down-regulated genes, six eggNOG/COG categories were over-represented. The over-represented categories corresponding to the lowest *p*-values in increasing order included the following: translation (J), energy production and conversion (C), cell wall/membrane/envelope biogenesis (M), intracellular trafficking and secretion (U), cell motility (N), and lipid transport and metabolism (I) (Fig. 5a). Among the 698 up-regulated genes, eight eggNOG/COG categories were over-represented. The over-represented categories with the lowest p-values in increasing order included the following: amino acid transport and metabolism (E), carbohydrate transport and metabolism (G), inorganic ion transport and metabolism (P), transcription (K), signal transduction mechanisms (T), energy production and conversion (C), coenzyme transport and metabolism (H), and secondary metabolite biosynthesis (Q) (Fig. 5b, Additional file 2: Table S2).

Introduction of *E. amylovora* cells to apple tree tissue also influenced the metabolic pathways of the bacteria. The genes of metabolic pathways in the general categories of metabolism, genetic information processing and environmental information processing were found among the differentially expressed genes. Among the up-regulated genes, over-representation of genes playing roles in the pathways of xenobiotic biodegradation and metabolism, signal transduction, energy and amino acid metabolism, and membrane transport was observed. On the other hand, among the down-regulated pathways, translation and transcription were mostly over-represented (Additional file 3: Table S3).

Out of all *E. amylovora* genes located on the chromosome and plasmid, in all experimental combinations, the highest abundance of transcripts was observed for the gene EAMY_3112, annotated to code a hypothetical protein, followed by the genes *ompA*, coding for outer membrane protein A precursor, and *lpp*, coding for a major outer membrane lipoprotein precursor. Out of 1633 genes that were differentially expressed (DEGs) in Idared 24 h after inoculation, expression of 288 genes (17.63%) was not found to be changed in Free Redstar. However, out of 1910 DEGs found in Free Redstar, 622 (32.56%) had unchanged expression in Idared. In the case of both apple cultivars, most down-regulated genes belonged to the group of genes responsible for siderophore biosynthesis, *dfoJAC*, and a few genes of glp regulon involved in glycerol catabolism. The most up-regulated gene in the two apple cultivars was *fldX;* in the case of Free Redstar, two other flavodoxin (electron-transfer protein) genes, *fldA3* and *fldZ*, were the most up-regulated. Among other highly up-regulated genes, some genes of the *ssuEADCB* cluster and gene *cbl* were found. The *ssu* genes are required for the utilization of sulfur from aliphatic sulfonates in *E. coli* and are regulated by the transcriptional regulator *cbl* [35]. Other genes that were significantly up-regulated included *cysGDN*, which also plays a role in sulfur metabolism. Among the most up- and down-regulated genes, several genes coding for hypothetical proteins were found.

The differences in expression of the most important genes involved in pathogenicity of *E. amylovora* were very similar between all combinations of pure bacterial culture vs. *in planta* transcriptomes. All or almost all genes involved in amylovoran biosynthesis, T3SS (hrp – PAI-1), sucrose and sorbitol metabolism, and biosynthesis of 6-thioguanine and c-di-GMP were up-regulated. Additionally, up-regulation *in planta* of a gene described on the genome of *E. amylovora* strain ATCC49946 as EAM_2938 and localized in the position 569,413 … 569,255 of the strain CFBP 1430 genome was observed. This gene, putatively coding for a membrane protein, was found by [36] to be up-regulated by *hrpL* – the alternative sigma factor that positively regulates transcription of T3SS components. Two sets of flagellar genes localized in different regions of genome (FlaI and FlaII), T3SS genes (PAI-2 and PAI-3), iron uptake genes (*foxR, dfoJAC*), and genes involved in T1SS - metalloprotease synthesis and secretion (*prtADEF*) were mostly down-regulated, or their expression was not differential (Additional file 4: Fig. S1). The main differences in expression change between pure bacterial culture vs. *in planta* were observed in FlaI genes. Almost all FlaI genes were down-regulated in Idared; half of these genes showed no change in the expression level in Free Redstar, and *fliOPQR* genes coding for inner membrane proteins involved in flagellar biosynthesis pathways were up-regulated, or their expression was not changed. Thiamin biosynthesis genes (*thiFGSO)* located on pEA29 plasmid were also up-regulated *in planta*.

In Free Redstar, up-regulation of the multidrug efflux pump genes *acrAB*, genes coding for components of other multidrug efflux pumps, such as *aaeA, mdtB, norM, emrAB3*, genes coding for some permeases involved in the transport of metabolites or resistance to toxic substances, such as *ydfJ, ydhC, ccmB, eamA, rhaT,* and *yrbE,* and several other membrane proteins was observed. Expression of these genes was not changed in Idared. Genes *srfABC*, annotated to be putative virulence factors and known to be responsible for the biosynthesis of surfactin, a surface cyclic lipopeptide in *Bacillus subtilis* [37], were down-regulated in Idared, but their expression was unchanged in Free Redstar.

We also tested the difference in expression of 40 sRNAs identified by Zeng et al. [11]. Differences in expression between bacterial culture and *in planta* were observed only for sRNA *gcvB* and hrs17. These two sRNAs were down-regulated in apple shoots (Additional file 5: Table S4).

The majority of the genes of the type VI secretion system T6SS – cluster 1 and cluster 3 [38] were down-regulated, or no expression changes were observed *in planta* compared to in pure bacterial culture, with the exception of the EAMY_3224 gene, coding for a membrane protein, whose expression was 2.66 and 3.99 times higher in Idared and Free Redstar, respectively*.* Genes of the T6SS – cluster 2 were up-regulated, or their expression was not changed.

### Difference in *E. amylovora* transcriptome response in apple trees of different susceptibility to fire blight 24 h after inoculation

At 24 h post inoculation, compared to the transcriptome of bacteria grown in TY medium, in Idared and Free Redstar, 1057 and 1020 genes were up-regulated, and 922 and 817 genes were down-regulated, respectively, (Table 1, Additional file 6: Table S5abcd). Among these genes, in Idared and Free Redstar, 162 and 125 were uniquely up-regulated, while 194 and 84 were uniquely down-regulated, respectively.

At 24 h after inoculation, compared to in Free Redstar, in Idared, 150 genes of *E. amylovora* 650 had significantly higher expression, and 142 genes had significantly lower expression (Additional file 7; Table S6, Additional file 8: Table S7). The most differentially expressed genes were those coding for hypothetical proteins, based on the fact that they are not classified to any COG/eggNOG category. Generally, genes coding for hypothetical, putative or uncharacterized proteins constituted half of all calculated DEGs.

For the COG/eggNOG categories that were differentially expressed in the two apple cultivars, the highest differences were observed for categories C (energy production and conversion), E (amino acid transport and metabolism - including genes involved in methionine biosynthetic process (*metAEFR*) and glycine cleavage genes *gcvTHP*) and G (carbohydrate transport and metabolism including glycogen *glgBXCAP* operon), which were uniquely or more often represented among genes of higher expression in Idared than in Free Redstar. Among categories of genes of higher expression in Free Redstar than in Idared, M (cell wall/membrane/envelope biogenesis), N (cell motility), O (posttranslational modification, protein turnover, chaperones) and V (defence mechanisms) were all prevalent.

The most differentially expressed *E. amylovora* genes between the two apple cultivars are annotated to code for hypothetical proteins. Gene EAMY_3203, which was found to be 80.64 times up-regulated in Idared compared to in Free Redstar, is placed on the *E. amylovora* CFBP 1430 genome in the group of genes annotated to code for hypothetical proteins and located between T6SS genes. The three most up-regulated genes in Free Redstar compared to in Idared were EAMY_0674, EAMY_0946, EAMY_2509, also coding for hypothetical proteins. Gene EAMY_0674 is located on the *E. amylovora* chromosome in the group of genes related to heme utilization or adhesion, while the functions of the rest of genes in this group are unknown. The other two genes are also located in groups of genes with unidentified functions.

Among *E. amylovora* genes with higher expression in Idared than in Free Redstar, genes involved in biotin synthesis, *bioABFCD* and *ynfK*, were found, as well as two genes possibly involved in adhesion, *csuB* and *csuE,* and one biofilm regulator *yceP*. Genes *yhcN1* and *yhcN3*, coding for members of the family of proteins functioning as acid stress adaptation factors in *Yersinia pestis* [39] or described as a biofilm modulation genes in *Escherichia coli* [40] were also more highly expressed in Idared than in Free Redstar, as well as the *ygiW* gene coding for a protein that is important in stress responses, including resistance to H_2_O_2_, cadmium and acid, and that is important in biofilm formation in *E. coli* [41], the *alsSD* gene, coding for a-acetolactate synthase/decarboxylase, and *yidQ*, which is described as a hyperadherence gene helping to colonize host tissue in the genome of *Pantoea ananatis* [42]. In Idared, there were also up-regulated genes known to function in cellular metabolism under anaerobic conditions, namely, *adhE1, sfsA, frdC,* and *yceJ* [43], as well as genes that code alcohol dehydrogenases, *yghA, adhB, adhP,* and *adhE1*, and *ftnA*, which codes ferritin, a universal protein that stores iron and releases it in a controlled fashion.

In Free Redstar, up-regulated genes included the *suhB* gene, which has not been studied in *E. amylovora* but has been found to be an essential gene for T3SS gene expression in *Pseudomonas aeruginosa* [44], *ampC*, which is one of the beta-lactamase coding genes localized in the *E. amylovora* genome, *inlA*, coding for a precursor of intenalin-A, a surface protein known to be used by *Listeria monocytogenes* to invade mammalian cells [45], and *srfABC*, surfactin biosynthesis genes [37].

In the genome of CFBP 1430, four *pqq* operon genes coding for pyrroloquinoline quinone (PQQ) were annotated (*pqqBCDE*). In this study, we found a fifth one, *pqqA*, located between positions 3,173,704 and 3,173,784 of the CFBP 1430 genome. Three of these *pqqECB* genes were found to be up-regulated in Free Redstar. PQQ is known to be a small, redox-active molecule that serves as a cofactor for several bacterial dehydrogenases, introducing pathways for carbon utilization that confer a growth advantage, but it was also shown to be essential for antibacterial activity of the biocontrol agent *Rahnella aquatilis* [46].

Among genes known to be important for the pathogenicity of *E. amylovora,* one of two sets of flagellar genes, FlaI, was found to be expressed more in Free Redstar 24 h after infection*.* In Free Redstar, 25 FlaI genes were up-regulated 24 h after inoculation compared to in Idared. A similar observation was made for ten chemotaxis, motility and biofilm formation genes located within FlaI (*cheZ1, cheY1, cheB1, cheR1, tap3, tsr3, cheW1, cheA1, notB1,* and *motA1*) and the gene *aer* located in another region of the genome.

We found other genes known to play or possibly play a role in pathogenicity that were more highly expressed in Idared, including *hrpA1*, belonging to T3SS, *galF*, a precursor of amylovoran formation, *srlA*, sorbitol permease*,* three (EAMY_1021, EAMY_1023, EAMY_1024) out of five genes involved in biosynthesis of 6-thioguanine and *argD*, a gene coding for the N-acetylornithine aminotransferase enzyme, which is involved in the production of the amino acid arginine, and mutation in this gene causes arginine auxotrophy, nonpathogenicity in apples, and reduced virulence in pears [47].

In Free Redstar, up-regulation of the multidrug efflux pump gene *acrA*, which is a part of the multidrug efflux pump AcrAB required for virulence of *E. amylovora*, resistance towards apple phytoalexins and successful colonization of the host plant [14, 16], was observed. Up-regulation was also noted for another set of multidrug efflux pumps, *emrA* and *emrB3*, protecting the cell from several chemically unrelated antimicrobial agents [48], and for genes *aaeA* and *aaeB*, which are subunits of the p-hydroxybenzoic acid efflux pump.

Among the two-component signal transduction system (TCST) genes present in the *E. amylovora* genome, differences in expression between two apple cultivars were observed only for genes related to the motility of bacterial cells: *cheA1, cheB1, cheY1*, which were up-regulated in Free Redstar, and *baeR*, which was down-regulated in this cultivar. *baeR* may play a role in the virulence of *E. amylovora* because its overexpression significantly increased amylovoran biosynthesis [49].

In Idared, we also observed higher expression of the *rmsA* (*csrA)* gene, which has been the subject of contradictory reports. Ancona et al. [50] used knock-out mutants to find that *rsmA* (*csrA*) positively regulates virulence factors, such as motility, amylovoran production, and T3SS, while Ma et al. [51] showed that the presence of many copies of the *rsmA* gene in an *E. amylovora* cell supresses motility and EPS production. The protein RsmA creates a regulatory system with a nontranslatable RNA regulator *rmsB (csrB).* Ancona et al. [50] and Ma et al. [51] showed opposite roles of *rmsB*. In studies by Ancona et al. [50], ΔcsrB mutants were hypermotile, overproduced EPS, and showed increased expression of T3SS, while according to Ma et al. [51], multiple copies of *rsmB* in *E. amylovora* cell induced the same effect. In our studies, no difference in expression of *rmsB* was observed between cultivars, but a generally smaller amount of *rmsB* was detected in *E. amylovora in planta* than in pure bacterial culture.

All stress-related genes in the CFBP 1430 genome had different expression *in planta* than in pure bacterial culture. Of these, 17 genes, whose products participate in stress responses, were identified to be differentially expressed in Idared and Free Redstar 24 h after infection. The increased expression in Free Redstar was observed for 11 genes: genes related to heat shock (*clpB3*, *dnaJ*, *dnaK*, *grpE, htrB, htpG, hslU, ibpA*, EAMY_0674) and cold shock (*deaD, cspA*), while in Idared increased expression was observed for only six genes: *cspD*, related to cold shock, *yedU*, related to heat shock, and genes playing roles in the general stress response: *dps, yfiA, A* and *uspB*. This result indicates that the environment in Free Redstar is more stressful for bacterial cells than that in Idared because a higher number of stress-related genes were more intensively expressed; primarily heat shock-related genes are involved in defence against stress.

Differences in expression of a few genes coding for outer membrane proteins were detected between Idared and Free Redstar. Outer membrane proteins create a selective barrier and protect the bacteria from the environment by preventing the entry of many toxic molecules into the cell; additionally, they are members of transport systems. Out of 307 genes in the *E. amylovora* CFBP 1430 genome annotated to code for membrane proteins, 24 were differentially expressed in the two apple cultivars, and 11 and 13 were up-regulated in Idared and in Free Redstar, respectively. Among membrane protein genes with higher expression in Idared, genes responsible for the transport of amino acids, inorganic ions and coenzymes and for envelope biogenesis were observed. The up-regulated membrane protein genes in Free Redstar included genes playing a role in intracellular trafficking and secretion, defence mechanisms, lipid and inorganic ion transport and metabolism, envelope biogenesis, and cell cycle control (Additional file 9: Table S8).

### Differences in expression of *E. amylovora* genes 24 h and 6 days after inoculation of Idared and free Redstar shoots

A similar number of *E. amylovora* genes were differentially expressed 24 h and 6 days after inoculation of Idared and Free Redstar shoots: 1013 and 1082, respectively. In both cases, about half of the DEGs were up-regulated and down-regulated (Table 1, Additional file 10: Table S9, Additional file 11: Table S10). Taking into consideration the COG/eggNOG categories, a different number of genes belonging to some of the categories were up-regulated and down-regulated in Idared compared to in Free Redstar (Additional file 12: Fig. S2). Among the up-regulated categories, carbohydrate transport and metabolism genes (G) and transcription genes (K) were over-represented in Free Redstar, while intracellular trafficking and secretion genes (U) were under-represented in Idared. Among the down-regulated categories, genes belonging to the cell motility category (N) were over-represented in Free Redstar and under-represented in Idared. Additionally, over-representation of the categories of energy production and conversion (C), amino acid, nucleotide, carbohydrate and lipid transport and metabolism (E, F, G, I) were observed in Idared, and cell wall/membrane/envelope biogenesis (M) in Free Redstar.

Three genes were up-regulated in Free Redstar but down-regulated in Idared at both time points: EAMY_0930, coding for ornithine utilization regulator, EAMY_1210, a putative ABC transport system, and EAMY_1750, coding for a putative flavoprotein monooxygenase. Differences were observed in the most down- and up-regulated genes in two apple cultivars. In Free Redstar, the gene *pagC* was the most down-regulated gene (166.36 times down-regulated), while on Idared, it was down-regulated only by 2.36 times. PagC protein is a well described *Enterobacteriaceae* virulence membrane protein belonging to the family Ail/OmpX/PagC/Lom. The members of this family are responsible for conferring resistance to complement-mediated killing, survival in macrophages, and adhesion and invasion of host cells in *Yersinia* and *Salmonella* strains [52]. These proteins could be important for virulence by neutralizing host defence mechanisms. Comparative genomic analysis revealed that the *pagC* gene is present in pathogenic *E. amylovora* and *E. pyrifoliae* but not in the non-pathogenic *E. tasmaniensis* [53]. Among the down-regulated genes in Free Redstar, several genes coding for hypothetical proteins were found, but no change in their expression was observed in Idared and vice versa. A similar observation was made for the groups of the most up-regulated genes in both cultivars – they consist mostly of genes coding for hypothetical proteins (Additional file 10: Table S9, Additional file 11: Table S10).

Differences in expression of virulence-related genes were observed between Idared and Free Redstar while comparing samples 24 h and 6 days after inoculation. Comparing the expression of genes at 6 days to that at 24 h after inoculation, a higher number of genes involved in amylovoran and metalloprotease biosynthesis was down-regulated in Idared than in Free Redstar. In Idared, the expression of the majority of FlaI genes and motility genes was similar at the two time points, while in Free Redstar, more genes were down-regulated 6 days after infection compared to 24 h; no expression differences between apple cultivars were observed 6 days after inoculation. Two genes (*edcB* and *edcE*) involved in c-di-GMP biosynthesis were up-regulated in Idared, while in Free Redstar, their expression was not different between the two time points after inoculation. Almost all *hrp* T3SS genes were down-regulated at 6 days compared to at 24 h after inoculation, except the T3E avirulence gene *avrRpt2*, whose expression increased over time in both apple cultivars (Additional file 10: Table S9, Additional file 11: Table S10, Additional file 13: Fig. S3).

No change in expression was observed for genes of inv./spa-type T3SSs PAI-2, with the exception of two genes, *spaK* and *spaM1*, which were significantly down-regulated in Free Redstar 6 days after inoculation compared to at 24 h (11.33 and 48.67 times, respectively)*.* Similarly, for T3SS PAI-3, *prgK3* and *spaN3* genes were down-regulated in Free Redstar (fold changes of 3.09 and 73.55, respectively), while in Idared, over time, the *invB* gene was up-regulated, and the *sipC3* gene was down-regulated. The *srfABC* gene involved in surfactin biosynthesis, the multidrug efflux pump *acrAB* and the majority of chemotaxis and motility genes were down-regulated in Free Redstar while no expression changes were observed in Idared between the two time-points of the experiment.

### Differences in expression of *E. amylovora* genes in Idared and free Redstar 6 days after inoculation

Only 11 genes were found to be differentially expressed in the two apple cultivars 6 days after inoculation. In Idared, five genes were up-regulated compared to in Free Redstar: siderophore biosynthetic genes *dfoJAC,* one gene coding for the hypothetical protein EAMY_1906, the gene EAM_2938, which is newly annotated in the CFBP 1430 genome and known to contribute to the virulence of *E. amylovora* [36] (Additional file 5: Table S4, Additional file 14: Table S11).

In Free Redstar, six genes were up-regulated: *vanA*, a putative vanillate O-demethylase oxygenase subunit, EAMY_1750, a putative flavoprotein monooxygenase, *pucI*, a putative NCS1-family allantoin permease, and three hypothetical proteins, EAMY_1683, EAMY_1948 and EAMY_3440 (Additional file 14: Table S11). The VanA protein sequence of *E. amylovora* showed over 80% similarity to proteins in human and animal pathogens of the *Enterobacteriaceae* family. In these pathogens, VanA and VanB are responsible for resistance to glycopeptides – a group of antimicrobial compounds [54] while *pucI* is a gene coding for an allantoin transport protein. Allantoin is a naturally occurring compound and a major metabolic intermediate in most living organisms, including bacteria; it often accumulates in stressed plants and may also activate stress responses [55].

## Discussion

We used RNA-seq technology to analyse differences in the transcriptome of the lowly virulent *E. amylovora* strain 650 in apple shoots of two apple cultivars differing in their susceptibility to fire blight. The susceptible cultivar, Idared, could be easily infected by this strain, while the resistant one, Free Redstar, exhibited almost no disease symptoms after inoculation. The results of transcriptome analysis show clear differences between *E. amylovora* gene expression in the two apple cultivars. However, the only significant differences in expression of previously recognized genes crucial for pathogenesis were observed for flagellar genes (FlaI), which had higher expression in Free Redstar, and *hrpA,* three out of five genes involved in the biosynthesis of 6-thioguanine, which were more intensively expressed in Idared 24 h after inoculation. Six days after inoculation, siderophore biosynthetic genes *dfoJAC* were up-regulated in Idared.

The transcriptome analysis showed that expression of two sets of flagellar genes located in the *E. amylovora* genome, FlaI and FlaII, was differentially regulated. Compared to in the bacterial culture, the majority of FlaI genes were down-regulated *in planta*; no change in expression was observed for the majority of FlaII genes. From the studies of Zhao et al. [56], it is known that operon deletion of FlaII does not influence the motility of the tested strain. Moreover, a phylogenetic analysis based on concatenation of 14 conserved flagellar protein sequences revealed that both FlaI and FlaII are clustered with enterobacteria, but the phylogenetic position of the FlaI system is much closer to the phylogeny of *E. amylovora* species than that of FlaII, which is more closely related to those of *Sodalis glossinidius* – an insect endosymbiont [57]. The same phylogenetic origin was found for two non-flagellar T3SS pathogenicity islands, PAI-2 and PAI3, which were mostly down-regulated *in planta* in our studies, in contrast to hrp T3SS. However, they were previously reported to be uninvolved in *E. amylovora* virulence in plants [58] but involved in insect cell invasion by *S. glossinidius* [57]. These results indicate that PAI2, PAI3 and FlaII may be acquired from the same source by horizontal gene transfer [58].

Flagellum-based motility is important for the virulence of bacterial pathogens. In our experiment, we observed general down-regulation of FlaI genes *in planta* in Idared 24 h after inoculation and in both cultivars 6 days after inoculation compared to in bacterial culture. This is in agreement with the observations of Raymundo and Ries [59], who found that *E. amylovora* cells isolated directly from apple shoots are not motile. However, almost all FlaI genes were up-regulated 24 h after inoculation in Free Redstar compared to in Idared. The higher expression of *E. amylovora* flagellar genes in Free Redstar can explain why strain 650 cannot effectively attack Free Redstar trees. The conserved part of the flagellin polypeptide, the flg22-domain, which faces the inside of the flagellar tube, is recognized as PAMP (pathogen-associated molecular pattern) by the innate immune systems of plants [60], and as was found in the proteomic studies performed by Holtappels et al. [61], lower virulent strains have more flagellin- and motility-associated proteins. However, the question is why is the expression of *E. amylovora* flagellar genes higher in a resistant apple cultivar than in a susceptible one? Flagellum synthesis undergoes transcriptional and posttranscriptional regulation. At the transcriptional level, genes involved in flagellum synthesis are expressed in a hierarchical fashion. At the top of this hierarchy is the master regulator *flhDC*, as reviewed by Chilcott et al. [62]. At 24 h after inoculation, we observed that expression of *flhC1* was higher in Free Redstar, while the expression of *flhD1* was unchanged. The operon *flhDC* is sensitive to environmental and cell state sensors and is controlled by numerous regulators, including cAMP-CRP, H-NS, *EnvZ/OmpR*, *barA/uvrY* (*gacA/gacS*), *lrhA* and the phosphorelay system RcsCDB [6, 63], but these genes were not differentially expressed between the two apple genotypes. However, their levels of expression are unlikely to reflect the type of environmental signals they sense, which may be different between two apple cultivars. The second level of flagellar gene expression regulation includes the positive regulator σ^28^ factor encoded by the *fliA1* gene and a negative one, anti-σ^28^ protein, coded by *flgM.* We observed an up-regulation of *fliA1* in Free Redstar and no difference in expression of *flgM* between the two apple cultivars 24 h after inoculation, which can explain the higher expression of flagella synthesis genes in Free Redstar. Comparing expression of regulation genes 24 h and 6 days after inoculation, *fliA1* was down-regulated in both cultivars, and *flgM* was up-regulated in both cultivars at the later time point, while the *flhC1* gene was down-regulated in Free Redstar 6 days after inoculation, resulting in down-regulation of the majority of FlaI genes.

One of the clear differences in transcription of *E. amylovora* genes between the two apple genotypes was the transcription of stress-related genes. They were generally more highly expressed in the Free Redstar cultivar; most products were classified as heat shock proteins, which are a group of proteins that repress the denaturation of molecules by various stressful circumstances, such as heat, cold, UV light, oxygen, and Ca2+. A difference in abundance of these proteins was also observed among lowly and highly virulent *E. amylovora* strains based on proteomic studies performed on the leaves of a susceptible apple clone. More heat shock proteins were produced by a more virulent strain in a susceptible apple cultivar [64], while in the case of our study, the same was observed for a lowly virulent strain in a resistant cultivar. This type of protein was also induced during *E. amylovora* infection of immature pears [65].

Another group of genes that were more highly expressed in Free Redstar are genes of multidrug efflux pumps and permeases involved in the transport of metabolites or resistance to toxic substances. During infection of the plant, bacteria are exposed to a variety of antimicrobial compounds produced by the host; these protein structures are able to recognize and efficiently expel a wide range of structurally diverse compounds from the bacterial cell and play a very important role in the success of the pathogen [66]. This observation could suggest that Free Redstar produces more antimicrobial compounds, and therefore, expression of genes coding for proteins involved in detoxification of bacterial cells is higher in the more resistant cultivar.

Several genes, such as surfactin biosynthesis genes *srfABC, csuBE*, involved in adhesion, the biofilm regulator *yceP, suhB*, found to be important for T3SS in *P. aeruginosa,* and stress response genes that have not yet been shown to play a role in the pathogenic abilities of *E. amylovora* were found to be differentially expressed in the two apple cultivars. Based on this fact and on their function, structure or reports of their roles in other bacterial species, detailed studies are required to elucidate their role in the pathogenicity of *E. amylovora*. One of the most significant observations during this study is the fact that among genes with the highest fold change in expression between experimental combinations or the highest transcript abundance, there are several genes without ascribed functions. This fact suggests that although their role is unknown, their function could be important during interactions with a host plant. The importance of genes coding for hypothetical proteins was observed even during a study with a minimal cell concept, where an experimental design of a minimal synthetic genome revealed a surprising number of genes of unknown function – ca. 30% of the genome essential for bacterial life [67]. This is the general problem in genomics. At present, numerous genome projects are adding thousands of nucleotide sequences to public databases each day. The challenge is in translating sequence into function. The most common approach is to search databases for well-characterized proteins that have similar amino acid sequences to the protein encoded by a new gene and employ a method to explore the gene’s function from there. Using this approach, only a fraction of predicted genes will have annotated products and functions. In the genome of *E. amylovora* CFBP1430, over 850 predicted genes are annotated to be putative proteins, proteins of unknown function or hypothetical proteins. Approximately 40% of genes cannot be classified to any COG category or are classified to category S: function unknown. This is related to the fact that although studies on genes and their function have been conducted for many years by many teams, even using new challenging techniques, there is still much work ahead for scientists to fully understand all the processes in bacterial and eukaryotic cells. Additionally, some mistakes can be generated in RNA-seq data analyses because of weak points of the algorithms applied for data normalization and gene expression fold change calculation [68]. Particularly for the extremal values of fold change, differences can be observed depending on the algorithm applied; genes of particular interest should be additionally analysed with other techniques, e.g., real-time PCR.

Only a few genes were differentially expressed 6 days after inoculation in two apple cultivars, although clear differences in disease symptoms were observed. On Idared, four genes of known relation to pathogenicity were up-regulated – genes coding for siderophore desferrioxamine biosynthesis, *dfoJAC* and EAM_2938. Desferrioxamine plays a dual role in iron acquisition and protecting bacterial cells against lethal doses of hydrogen peroxide [19]. In Idared, extracellular development of the pathogen and a rapid host cell death likely lead to iron deficiency or higher antimicrobial activity of reactive oxygen species (ROS) because ROS are closely associated with lesion development after inoculation of apple leaves with *E. amylovora* [30]. In Free Redstar, genes known to play a role in stress responses and bacterial cell defence in other bacterial species were more intensively expressed, as well as a group of genes coding for proteins of unknown function.

Zhao et al. [65] identified *E. amylovora* genes induced during infection of immature pear tissue. We found that only approximately 30% of up-regulated genes listed by Zhao et al. [65] were also up-regulated in our tests on both apple cultivars. The different plant tissue, different experimental conditions, e.g., the microbiological medium used to grow bacteria prior to the inoculation, or different types of techniques used for gene expression analysis may be the reason for these discrepancies.

To elucidate the background of the differences in virulence of *E. amylovora* strains, Holtappels et al. [64] applied a proteomics approach. After separate inoculations of apple leaves with highly and lowly virulent strains, they identified a group of 154 proteins that were differentially expressed in these two tested strains. Only a few genes coding for these proteins were found to be differentially expressed in Idared and Free Redstar; proteins identified by Holtappels et al. [64] to be more abundant in lowly virulent strain were not found to be down-regulated in the more sensitive apple cultivar or vice versa. Out of the proteins that were more abundant in the lower virulence strain, four (*mdh, yedU, dps, yfiA*) were up-regulated, and three were down-regulated (*htpG, grpE, cspA*), while among the proteins that were more highly expressed in the more virulent strain, four (*argD, mdh, galF*, EAMY_3259) were up-regulated, and three were down-regulated (*dnaK, clpB3, rho*) in Idared compared to in Free Redstar 24 h after inoculation. However, as shown by Hack [69], the proteome and transcriptome data are quite often contradictory. The poor correlation between of mRNA and protein amounts is considered as a result of different factors. One of them is weak complementarity between Shine Dalgarno sequence on the transcript and rRNA what results in lower translation level. Additionally there is important role of a secondary structure of RNA, which can be changed in certain conditions and also influences the efficiency of translation [70]. Regulatory proteins [71] and sRNA [72] which act as translational modulators as well as other factors like half-life of protein, its location and interaction with other proteins play also a role in the efficiency of translation [73].

## Conclusions

This RNA-seq analysis generated a novel dataset describing the transcriptional response of the lowly virulent strain of *E. amylovora* in susceptible and resistant apple cultivar. The genes known as important for the *E. amylovora* pathogenicity were only slightly differentially expressed between apple cultivars. However, the higher expression of *E. amylovora* flagellar genes (recognized as PAMP) in Free Redstar can explain why strain 650 cannot effectively attack Free Redstar trees. Also higher expression of stress related genes and genes of multidrug efflux pumps and permeases can suggest that the environment in Free Redstar is more stressful for bacteria what can be the barrier for the efficient infection of this cultivar. Among genes with the highest fold change in expression between experimental combinations or with the highest transcript abundance, there are many genes without ascribed functions, which have never been tested for their role in pathogenicity. This fact suggests that although their role is unknown, their function could be important during interactions with a host plant.

## Methods

### *E. amylovora* virulence test

Strain 650 was isolated from a hawthorn with fire blight symptoms in central Poland and kept in the collection of the Laboratory of Bacteriology at the Research Institute of Horticulture, Skierniewice, Poland. To check its virulence, the test on apple cultivars of different susceptibility was performed. The shoots of three apple genotypes: Idared (susceptible), Elstar (middle susceptible) and Free Redstar (resistant), were used for inoculation by shoot tip cutting with scissors immersed in bacterial solution of strain 650. Fifteen trees were tested for each genotype. The virulence of *E. amylovora* strains was expressed as a percentage of shoot necrosis in relation to the entire length of the shoot measured 6 weeks after inoculation. The results were analysed with ANOVA, and means were separated with Tukey’s test at *P* = 0.05.

### Sample collection and RNA isolation

**Fig. 6:**
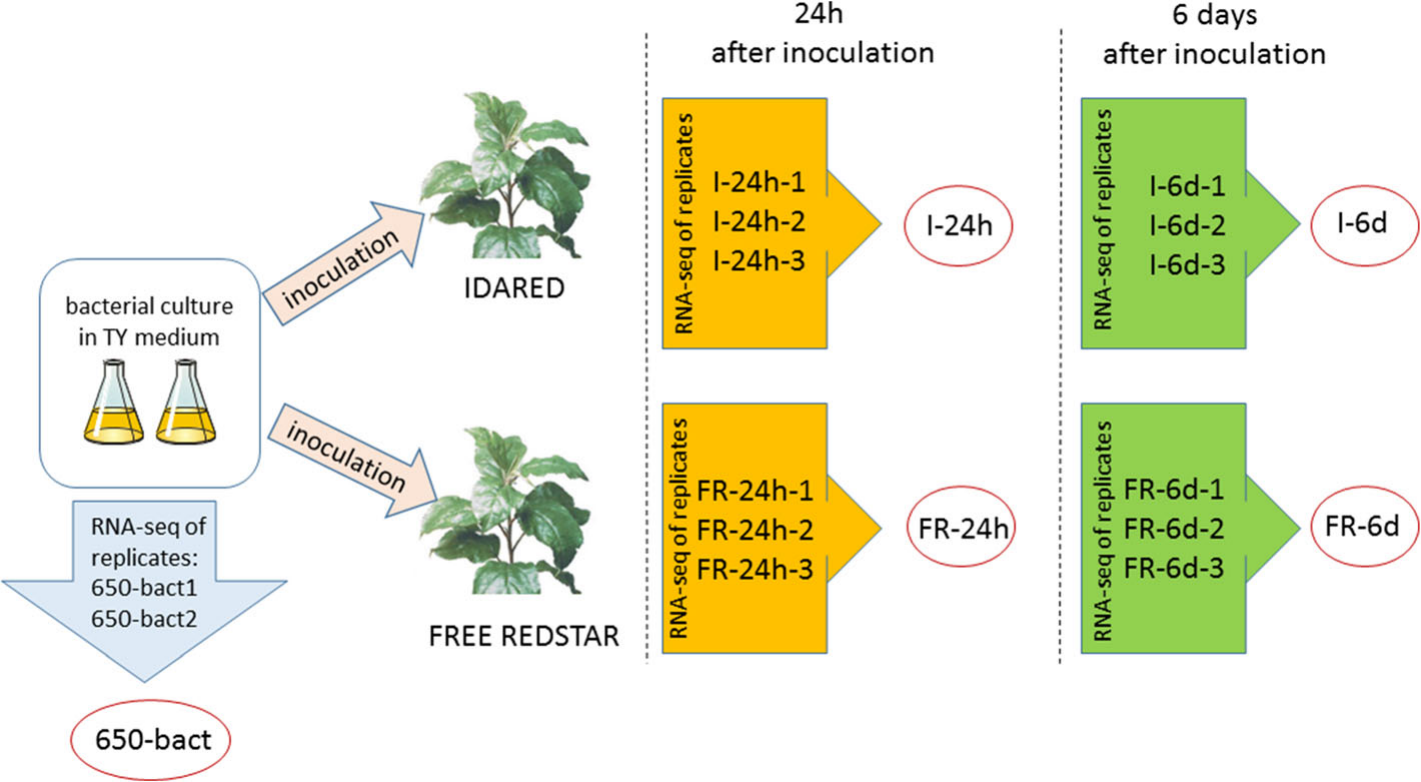
Diagram showing the study design. One-year-old, potted apple trees cultivars Idared and Free Redstar grafted on M.26 were inoculated with *E. amylovora* strain 650 in greenhouse conditions. Inoculation was made on actively growing shoots, with bacterial suspension grown overnight in TY medium. After 24 h and 6 days from the inoculation time total RNA was isolated from inoculated shoots. At each time point, RNA was isolated separately from at least 6 shoots of each apple cultivar. Additionally, RNA was isolated from the pure culture of *E. amylovora* 650 grown overnight in TY medium. In each time point, RNA was isolated from 3 biological replicates (in case of pure bacterial culture - two biological replicates). The replicates constituted the sample (marked by red circle)

One-year-old, potted apple trees cultivars Idared and Free Redstar grafted on M.26 were inoculated with *E. amylovora* strain 650 in greenhouse conditions. Inoculation was made on actively growing shoots, punctured with a sterile needle on approximately 7 cm of their length and covered by droplets of bacterial suspension grown overnight in TY (Bacto Tryptone 0.5%, Yeast Extract 0.3%, CaCl_2_ 0.065%) medium. After 24 h and 6 days from the inoculation time (Fig. 6), samples were processed, and total RNA was isolated with a Total RNA Purification Kit (Norgen Biotek), as described by Kałużna et al. [74]. At each time point, RNA was isolated separately from at least six shoots of each apple cultivar. Additionally, RNA was isolated from the pure culture of *E. amylovora* 650 grown overnight in TY medium – the same used for bacterial growth for inoculation purposes. DNA was removed from samples by DNAse treatment (Deoxyribonuclease I, ThermoScientific, Lithuania). The efficiency of DNA removal was tested by nested-PCR with the primers peant1/peant2 and AJ75/AJ76 [75, 76] complementary to plasmid pEA29. Determination of the quality and concentration of obtained RNA free from DNA was tested on an Agilent 2100 Bioanalyzer using the Agilent RNA 6000 Nano Kit according to the manufacturer’s instructions. Three samples of the best quality (RIN) of each apple cultivar were subjected to rRNA depletion using a Ribo-Zero™ Magnetic Kit (Gram-Negative Bacteria); they constituted three biological replicates for each experimental combination.

### Library preparation and sequencing

The rRNA depleted sample concentration was measured using the 2100 Bioanalyzer (Agilent) and an RNA 6000 Pico Kit (Agilent, 5067-1513). Since the RNA concentration was low, the maximum allowable volume (6 μl) was used for the library construction using NEBNext Ultra Directional RNA Library Preparation Kit for Illumina (New England Biolabs, E7420S). The libraries have been sequenced on the MiSeq (Illumina) using the MiSeq Reagent Kit v2 (500-cycles) (Illumina, MS-102-2003) in the PE250 read mode. The resulting reads were additionally trimmed with Cutadapt [77]. These sequence data have been submitted to the ArrayExpress (EMBL) databases under accession number E-MTAB-5630.

### Bioinformatic analysis

Low quality sequence ends (ambiguous base limit: 2, quality limit: 0.05) were trimmed using CLC Genomics Workbench (v. 8.1) (Qiagen) Trim Sequences tool. For mapping, *E. amylovora* strain CFBP 1430 genome (FN434113, FN434114), which consists of chromosome (3,805,573 bp) and pEA29 plasmid (28,259 bp) and carries 3706 and 28 CDS on chromosome and plasmid, respectively [35] was used. High quality sequences were aligned to this genome using the CLC RNA-seq reference mapping algorithm with settings appropriate for Prokaryotic genomes (mapping to gene regions only). A quality control to check whether the overall variability of the samples reflected their grouping and the reproducibility between repetitions was performed with Principal Component Analysis (PCA). For the differentially expressed gene (DEG) analysis, expression values were normalized using the Trimmed Mean of M values (TMM) [78], and DEGs were analysed using the Empirical Analysis of DGE tool based on Exact Test incorporated in the EdgeR Bioconductor package and implemented in CLC Genomics Workbench. A gene was considered to be differentially regulated between two conditions when the gene showed a total read number larger than five, a > 1.5-fold absolute fold-change ratio and an FDR-adjusted *p* value <0.05.

For the newly annotated genes and non-coding RNAs, the expression values were normalized as a percent of total reads mapped to the *E. amylovora* CFBP 1430 genome. The results were subjected to ANOVA, and significance of differences between means were tested using the Newman-Keuls test at *p* < 0.05.

Gene annotation based on Gene Ontology (GO) terms in biological process, molecular function and cellular component categories for the *E. amylovora* CFBP 1430 coding sequences were downloaded from the UniProt database (http://www.uniprot.org). To summarize the pathway information protein sequence, fasta files were submitted to KAAS (KEGG automatic annotation server) [79], and KEGG orthology assignments were obtained (Additional file 15: Table S12). The eggNOG 4.5 database [80] was used to annotate genes with common denominators or functional categories (i.e., derived from the original COG categories). Enrichment of COG and KEGG terms was evaluated by a hypergeometric distribution at FDR < 0.05. FDR derived significance thresholds was calculated using classical one-stage method [81].

### qPCR validation

Transcription expression reported in the present study was validated through real time PCR using 11 candidate genes selected out of most up-regulated, down-regulated and of similar expression genes comparing different experimental combination *in planta* to in TY medium. Real time PCR was conducted with newly designed primers (Additional file 16: Table S13). Herein, three biological replicates were used to evaluate the transcription expression of *E. amylovora* strain 650 in each apple cultivar at each time point. For gene amplification, total RNA was isolated, reverse transcribed and amplified with real-time PCR, as described by Kałużna et al. [82] The qPCR runs were performed on a Bio-Rad CFX96 thermocycler with SsoAdvanced SYBR Green Supermix (Bio-Rad, Hercules, CA) under the conditions described by Kałużna et al. [82] using the comparative 2 − ΔΔCt method.

Three genes previously reported to be the most stable in expression were used for normalization of RT-qPCR expression analysis of tested genes: *proC* (DNA-directed RNA polymerase subunit beta)*, recA* (recombinase A)*,* and *ffh* (signal recognition particle protein) [82]. For assessment of the association between RNA-seq and RT-qPCR, the Pearson’s correlation method was used.

## Additional files

Additional file 1: Table S1. Summary of RNA-seq data. (DOCX 15 kb)

Additional file 2: Table S2. Enriched COG/eggNOG categories among commonly up- and down-regulated genes of *E. amylovora in planta* vs. in pure bacterial culture (DOCX 14 kb)

Additional file 3: Table S3. Enriched secondary KEGG pathways among commonly up- and down-regulated genes of *E. amylovora in planta* vs. in pure bacterial culture (DOCX 14 kb)

Additional file 4: Fig. S1. Change of expression of known genes involved in pathogenicity of *Erwinia amylovora* between bacteria in TY medium (650-bact) and *in planta* (I-24 h and FR-24 h) 24 h after inoculation. (PDF 339 kb)

Additional file 5: Table S4. Expression of newly annotated and non-coding RNAs (DOCX 19 kb)

Additional file 6: Table S5. Differentially expressed genes of *E. amylovora* 650 between bacterial culture in TY medium(650-bact) and in apple shoots in two time points after inoculation (I-24 h, I-6d, FR-24 h, FR-6d). (XLSX 401 kb)

Additional file 7: Table S6. *Erwinia amylovora* 650 genes up-regulated in Free Redstar (FR-24 h) in comparison to Idared (I-24 h) 24 h after inoculation (XLSX 23 kb)

Additional file 8: Table S7. *Erwinia amylovora* 650 genes up-regulated in Idared (I-24 h) in comparison to Free Redstar (FR-24 h) 24 h after inoculation (XLSX 23 kb)

Additional file 9: Table S8. Genes of *E. amylovora* 650 coding for membrane proteins and differentially expressed in two apple cultivars – Idared and Free Redstar (I-24 h and FR-24 h) 24 h after inoculation. (DOCX 16 kb)

Additional file 10: Table S9. Differentially expressed genes of *E. amylovora* 650 genes between two time point of experiment - 24 h (FR-24 h) and 6 days (FR-6d) after inoculation of Free Redstar shoots (XLSX 121 kb)

Additional file 11: Table S10. Differentially expressed genes of *E. amylovora* 650 genes between two time point of experiment - 24 h (I-24 h) and 6 days (I-6d) after inoculation of Idared shoots (XLSX 111 kb)

Additional file 12: Fig. S2. The content of different COG/eggnog categories among genes of different expression between two time points after inoculation–24 h and 6 days. UP –up-regulated, DOWN –down-regulated, I –Idared, FR –Free Redstar, 6d −6 days. (PDF 122 kb)

Additional file 13: Fig. S3. Change of expression of known genes involved in pathogenicityof Erwinia amylovorabetween24 h (FR-24 h) and 6 days (FR-6d) after inoculation on Idared and on Free Redstar (PDF 322 kb)

Additional file 14: Table S11. *Erwinia amylovora* 650 genes differentially regulated in Free Redstar (FR-6d) and in Idared (I-6d) 6 days after inoculation (XLSX 12 kb)

Additional file 15: Table S12. KEGG orthology assignments for genes located on *E. amylovora* CFBP 1430 genome (XLSX 121 kb)

Additional file 16: Table S13. Primers used for qRT-PCR validation of RNAseq data. (DOCX 14 kb)

## Abbreviations

ANOVA: Analysis of variance
COG: Clusters of Orthologous Groups
DEGs: Differentially expressed genes
EPS: Exopolysaccharides
FDR: False discovery rate
GO: Gene onthology
KAAS: KEGG automatic annotation server,
KEGG: Kyoto encyclopedia of genes and genomes
PAL: Phenylalanine ammonia lyase
PAMP: Pathogen-associated molecular pattern
PCA: Principal component analysis
PQQ: Pyrroloquinoline quinone
QTL: Quantitative trait Loci
RT-qPCR: Reverse transcribed quantitative polymerase chain reaction
SNP: Single nucleotide polymorphism
T3E: Type 3 effector
T3SS: Type 3 secretion system
T6SS: Type 6 secretion system
TCSTs: Two component signal transduction systems
TMM: Trimmed mean of m values
TY: Tryptone yeast
VPE: Vacuolar processing enzyme
