# Supplementary material for: Comparative transcriptome analysis of a lowly virulent strain of Erwinia amylovora in shoots of two apple cultivars – susceptible and resistant to fire blight
Source: BMC Genomics. 2017 Nov 13;18:868. doi: 10.1186/s12864-017-4251-z (PMC5683332; doi:10.1186/s12864-017-4251-z)
Supplement: Supplementary file 1 — Summary of RNA-seq data. (DOCX 15 kb) [file 12864_2017_4251_MOESM1_ESM.docx]

| Sample^1^ | Total reads | Mapped reads to *Ea* CFBP1430 | % of reads that aligned to rRNA operon | Mapping to genes^2^ | | | | |
| --- | --- | --- | --- | --- | --- | --- | --- | --- |
|  |  |  |  | Mapped reads to genes | Mean read length | Total read length | Unique match | Multi-position match |
| 650-bact-1 | 5 370 322 | 5 324 585 | 0.24 | 4 324 683 | 175.57 | 759 291 863 | 4 298 219 | 26 464 |
| 650-bact-2 | 6 523 562 | 6 473 977 | 0.30 | 5 220 697 | 173.23 | 904 356 299 | 5 185 916 | 34 785 |
| I-24h-1 | 8 421 680 | 6 265 973 | 0.44 | 4 836 922 | 162.43 | 785 661 234 | 4 798 812 | 38 130 |
| I-24h-2 | 17 099 456 | 13 106 400 | 0.35 | 10 215 328 | 180.94 | 1 848 406 962 | 10 156 672 | 58692 |
| I-24h-3 | 11 818 412 | 8 241 906 | 0.23 | 6 219 599 | 179.88 | 1 118 780 163 | 6 187 662 | 31 971 |
| I-6d-1 | 6 409 652 | 5 971 401 | 0.31 | 3 440 334 | 176.80 | 608 255 810 | 3 408 192 | 32 150 |
| I--6d-2 | 5 989 272 | 5 623 448 | 0.92 | 3 928 508 | 162.65 | 638 956 363 | 3 902 657 | 25 855 |
| I-6d-3 | 5 282 106 | 4 853 828 | 0.29 | 2 792 111 | 151.10 | 421 889 591 | 2 746 183 | 45 928 |
| FR-24h-1 | 6 374 298 | 4 944 730 | 0.38 | 3 828 867 | 165.74 | 634 601 181 | 3 792 077 | 36 800 |
| FR-24h-2 | 15 133 816 | 10 158 692 | 0.94 | 2 357 124 | 172.98 | 407 736 404 | 2 339 885 | 17 253 |
| FR-24h-3 | 9 822 778 | 5 644 781 | 0.40 | 4 414 825 | 153.12 | 675 984 871 | 4 379 570 | 35 257 |
| FR-6d-1 | 4 628 510 | 3 659 426 | 0.33 | 2 075 683 | 138.44 | 287 353 072 | 2 043 861 | 31834 |
| FR-6d-2 | 9 154 674 | 7 036 188 | 0.32 | 3 519 671 | 150.78 | 530 704 226 | 3 468 087 | 51 588 |
| FR-6d-3^3^ | 5 886 958 | 4 607 201 | 72.95 | 1 320 224 | 152.80 | 201 733 704 | 173 075 | 1 147 149 |

Table S1. Summary of RNA-seq data.

1. 650 – *E. amylovora* strain used in the study; bact – RNA isolated from pure bacterial culture; I – Idared; FR – Free Redstar; 24h – sample collected 24 h after inoculation; 6d - sample collected 6 days after inoculation; 1 or 2 or 3 – number of biological replicate.
2. Used in RNA-seq analysis.
3. Sample eliminated from transcriptome analysis
