# Supplementary material for: Comparative transcriptome analysis of a lowly virulent strain of Erwinia amylovora in shoots of two apple cultivars – susceptible and resistant to fire blight
Source: BMC Genomics. 2017 Nov 13;18:868. doi: 10.1186/s12864-017-4251-z (PMC5683332; doi:10.1186/s12864-017-4251-z)
Supplement: Supplementary file 2 — Enriched COG/eggNOG categories among commonly up- and down-regulated genes of E. amylovora in planta vs. in pure bacterial culture (DOCX 14 kb) [file 12864_2017_4251_MOESM2_ESM.docx]

| **COG/eggNOG** | **Enrichment ratio*** | **FDR-derived  p-values** |
| --- | --- | --- |
| Over-represented COGs among 640 down regulated genes | | |
| J - translation | 3.27 | 3.624E-30 |
| C - energy production and conversion | 2.11 | 3.061E-07 |
| M - cell wall/membrane/envelope biogenesis | 1.62 | 1.909E-04 |
| I - lipid transport and metabolism | 1.51 | 0.047 |
| Under-represented COGs among 640 down regulated genes | | |
| \| S – function unknown \| \| --- \| | 0.52 | 3.426E-29 |
| \| H - coenzyme transport and metabolism \| \| --- \| | 0.60 | 0.033 |
| \| K - transcription \| \| --- \| | 0.73 | 0.033 |
| \| E - amino acid transport and metabolism \| \| --- \| | 0.79 | 0.033 |
| Over-represented COGs among 698 up regulated genes | | |
| E - amino acid transport and metabolism | 1.85 | 3.863E-12 |
| G - carbohydrate transport and metabolism | 1.73 | 1.979E-06 |
| P - inorganic ion transport and metabolism | 1.50 | 1.293E-04 |
| K - transcription | 1.35 | 2.862E-03 |
| T - signal transduction mechanisms | 1.44 | 0.01 |
| C - energy production and conversion | 1.19 | 0.039 |
| H - coenzyme transport and metabolism | 1.21 | 0.039 |
| Q - secondary metabolites biosynthesis | 1.68 | 0.044 |
| Under-represented COGs among 698 up regulated genes | | |
| J - translation | 0.21 | 7.022E-07 |
| L - replication | 0.43 | 0.003 |
| S – function unknown | 0.80 | 0.005 |
| N - cell motility | 0.55 | 0.015 |
| D - cell cycle control, cell division and chromosome partitioning | 0.29 | 0.044 |
| O - posttranslational modification, protein turnover, chaperones | 0.63 | 0.044 |

Table S2. Enriched COG/eggNOG categories among commonly up- and down-regulated genes of *E. amylovora in planta* vs. in pure bacterial culture

*Enrichment ratio = proportion of COG X in the group of analysed set of *E. amylovora* differentially expressed genes/ proportion of COG X in *E. amylovora* genome
