# Supplementary material for: Comparative transcriptome analysis of a lowly virulent strain of Erwinia amylovora in shoots of two apple cultivars – susceptible and resistant to fire blight
Source: BMC Genomics. 2017 Nov 13;18:868. doi: 10.1186/s12864-017-4251-z (PMC5683332; doi:10.1186/s12864-017-4251-z)
Supplement: Supplementary file 3 — Enriched secondary KEGG pathways among commonly up- and down-regulated genes of E. amylovora in planta vs. in pure bacterial culture (DOCX 14 kb) [file 12864_2017_4251_MOESM3_ESM.docx]

| **Secondary KEGG pathway** | **Enrichment ratio*** | **FDR-derived  p-values** |
| --- | --- | --- |
| Over-represented KEGG pathways among 640 down regulated genes | | |
| Translation | 4.31 | 9.212E-37 |
| Energy metabolism | 1.86 | 5.547E-04 |
| Transcription | 5.07 | 0.007 |
| Under-represented KEGG pathways among 640 down regulated genes | | |
| Replication and repair | 0.35 | 0.002 |
| Carbohydrate metabolism | 0.78 | 0.022 |
| Metabolism of cofactors and vitamins | 0.66 | 0.030 |
| Amino acid metabolism | 0.67 | 0.007 |
| Membrane transport | 0.66 | 0.007 |
| Over-represented KEGG pathways among 698 up regulated genes | | |
| Amino acid metabolism | 1.40 | 0.005 |
| Xenobiotics biodegradation and metabolism | 2.38 | 0.005 |
| Membrane transport | 1.43 | 0.005 |
| Energy metabolism | 1.60 | 0.009 |
| Signal transduction | 1.54 | 0.012 |
| Under-represented KEGG pathways among 698 up regulated genes | | |
| Translation | 0.07 | 4.8E-05 |
| Replication and repair | 0.20 | 3.3E-04 |
| Folding, sorting and degradation | 0.23 | 0.009 |
| Nucleotide metabolism | 0.53 | 0.009 |

Table S3. Enriched secondary KEGG pathways among commonly up- and down-regulated genes of *E. amylovora in planta* vs. in pure bacterial culture

*Enrichment ratio = proportion of secondary KEGG pathway X in the group of analysed set of *E. amylovora* differentially expressed genes/ proportion of secondary KEGG pathway X in *E. amylovora* genome
