# Supplementary material for: Comparative transcriptome analysis of a lowly virulent strain of Erwinia amylovora in shoots of two apple cultivars – susceptible and resistant to fire blight
Source: BMC Genomics. 2017 Nov 13;18:868. doi: 10.1186/s12864-017-4251-z (PMC5683332; doi:10.1186/s12864-017-4251-z)
Supplement: Supplementary file 4 — Change of expression of known genes involved in pathogenicity of Erwinia amylovora between bacteria in TY medium (650-bact) and in planta (I-24 h and FR-24 h) 24 h after inoculation. (PDF 339 kb) [file 12864_2017_4251_MOESM4_ESM.pdf]

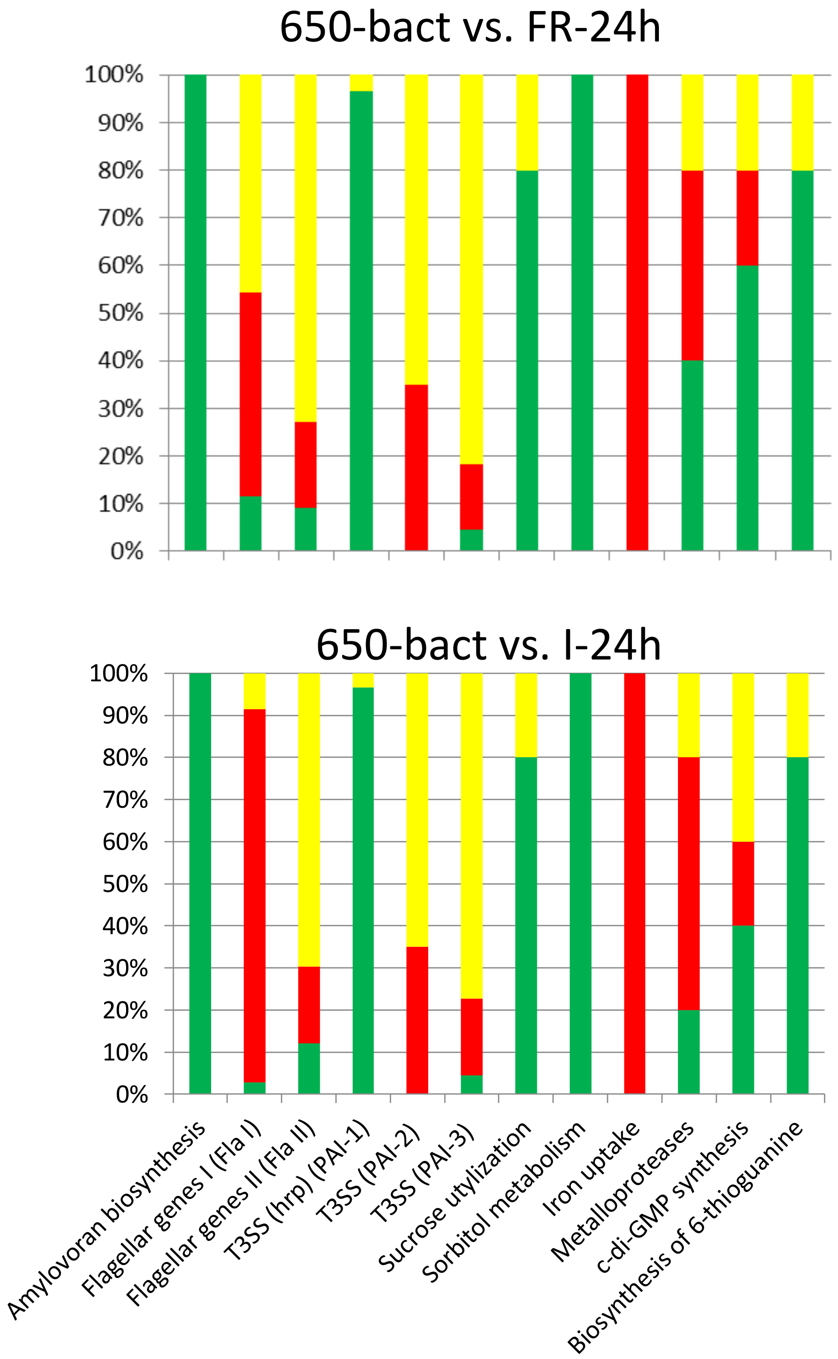

Fig. S1.
Change of expression of known genes involved in pathogenicity of *Erwinia amylovora* between bacteria in TY medium (650-bact) and 24 h after inoculation: I-24h on Idared and FR-24h on Free Redstar

NO CHANGE DOWN- UP-REGULATION *IN PLANTA*
