# Supplementary material for: Comparative transcriptome analysis of a lowly virulent strain of Erwinia amylovora in shoots of two apple cultivars – susceptible and resistant to fire blight
Source: BMC Genomics. 2017 Nov 13;18:868. doi: 10.1186/s12864-017-4251-z (PMC5683332; doi:10.1186/s12864-017-4251-z)
Supplement: Supplementary file 5 — Expression of newly annotated and non-coding RNAs (DOCX 19 kb) [file 12864_2017_4251_MOESM5_ESM.docx]

Table S4. Expression of newly annotated genes and non-coding RNAs

| Name | Position in CFBP1430 genome (FN434113) | 650-bact (TY culture) | I-24h | I-6d | FR-24h | FR-6d |
| --- | --- | --- | --- | --- | --- | --- |
| **Hfq-dependent sRNAs** | | | | | | |
| spf(spo42) | 52654 … 52816 | 6,58E-04^a*^ | 2,02E-03^a^ | 2,13E-03^a^ | 1,77E-03^a^ | 2,90E-03^a^ |
| hrs1 | 130071 … 130143 | 3,07E-05^a^ | 6,40E-05^a^ | 8,31E-05^a^ | 8,03E-05^a^ | 6,83E-05^a^ |
| hrs2 | 245013 … 245077 | 0^a^ | 1,04E-05^a^ | 1,12E-05^a^ | 0^a^ | 2,73E-05^a^ |
| hrs3 | 2936870 … 2936763 | 5,26E-05^a^ | 9,78E-05^a^ | 2,14E-04^a^ | 3,84E-05^a^ | 1,10E-04^a^ |
| micM(sroB) | 1111723 … 1111808 | 0^a^ | 1,76E-04^a^ | 1,52E-04^a^ | 1,72E-04^a^ | 0^a^ |
| hrs4 | 1214761 … 1214855 | 4,60E-05^a^ | 2,48E-04^a^ | 2,85E-04^a^ | 6,72E-04^a^ | 3,01E-04^a^ |
| hrs5 | 1370909 … 1371024 | 1,17E-04^a^ | 9,27E-05^a^ | 2,85E-04^a^ | 1,81E-04^a^ | 8,36E-05^a^ |
| hrs6(rmaA) | 1745925 … 1746022 | 2,50E-04^a^ | 2,59E-04^a^ | 1,34E-04^a^ | 2,02E-04^a^ | 1,40E-04^a^ |
| hrs7 | 1926536 … 1926641 | 4,93E-05^a^ | 4,36E-03^a^ | 1,36E-02^a^ | 2,25E-03^a^ | 7,90E-03^a^ |
| ryhB | 1944214 … 1944267 | 0^a^ | 0^a^ | 1,19E-05^a^ | 0^a^ | 0^a^ |
| hrs8 | 2095304 … 2095468 | 6,23E-04^a^ | 1,38E-03^a^ | 2,45E-03^a^ | 1,30E-03^a^ | 1,53E-03^a^ |
| hrs9 | 2277964 … 2278058 | 6,24E-04^a^ | 8,02E-04^a^ | 6,18E-04^a^ | 8,75E-04^a^ | 4,37E-04^a^ |
| hrs10 | 2319094 … 2319241 | 5,59E-03^a^ | 1,59E-02^a^ | 2,07E-02^a^ | 1,15E-02^a^ | 2,40E-02^a^ |
| hrs11 | 2361563 … 2361691 | 3,83E-05^a^ | 1,35E-04^a^ | 1,27E-04^a^ | 1,87E-04^a^ | 4,10E-05^a^ |
| hrs12 | 2400555 … 2400641 | 0^a^ | 1,02E-05^a^ | 0^a^ | 1,18E-05^a^ | 2,73E-05^a^ |
| hrs13 | 2573226 … 2573307 | 0^a^ | 1,32E-05^a^ | 1,19E-05^a^ | 4,89E-05^a^ | 5,47E-05^a^ |
| micA | 899250 … 899138 | 1,93E-03^a^ | 3,02E-03^a^ | 1,62E-03^a^ | 2,77E-03^a^ | 1,04E-03^a^ |
| gcvB | 808561 … 808353 | 2,57E-01^a^ | 3,23E-02^b^ | 5,25E-02^b^ | 2,97E-02^b^ | 1,05E-01^b^ |
| omrAB | 761953 … 761874 | 0^a^ | 0^a^ | 3,49E-05^a^ | 1,18E-05^a^ | 8,20E-05^a^ |
| arcZ(ryhA) | 371942 … 371739 | 6,46E-03^a^ | 1,77E-02^a^ | 1,24E-02^a^ | 1,18E-02^a^ | 6,33E-03^a^ |
| hrs15(ryhB) | 3573224 … 3573358 | 6,52E-05^a^ | 1,17E-04^a^ | 4,38E-04^a^ | 9,25E-05^a^ | 5,83E-04^a^ |
| hrs16 | 3790293 … 3790408 | 1,86E-05^a^ | 2,94E-05^a^ | 0^a^ | 4,30E-05^a^ | 1,42E-05^a^ |
| hrs17 | 83003 … 83246 | 8,49E-01^a^ | 2,13E-01^b^ | 1,83E-01^b^ | 1,18E-01^b^ | 3,76E-01^b^ |
| hrs18 | 118352 … 118269 | 3,72E-05^a^ | 4,72E-05^a^ | 2,79E-05^a^ | 4,01E-05^a^ | 2,84E-05^a^ |
| hrs19 | 1082386 … 1082451 | 1,53E-05^a^ | 3,19E-05^a^ | 1,37E-05^a^ | 0^a^ | 0^a^ |
| hrs20 | 1175035 … 1175114 | 1,86E-05^a^ | 1,33E-04^a^ | 7,58E-05^a^ | 3,04E-04^a^ | 2,05E-04^a^ |
| hrs21 | 1229904 … 1230037 | 6,06E-03^a^ | 4,93E-03^a^ | 1,73E-03^a^ | 4,64E-03^a^ | 3,04E-03^a^ |
| hrs23 | 1729503 … 1729640 | 3,39E-05^a^ | 3,63E-05^a^ | 4,72E-05^a^ | 2,53E-05^a^ | 1,42E-05^a^ |
| hrs24 | 1757245 … 1757335 | 1,14E-03^a^ | 7,08E-04^a^ | 9,91E-04^a^ | 9,21E-04^a^ | 1,16E-03^a^ |
| hrs25 | 1856397 … 1856485 | 0^a^ | 2,13E-05^a^ | 1,12E-05^a^ | 1,18E-05^a^ | 0^a^ |
| hrs26 | 1872230 … 1872339 | 9,86E-05^a^ | 1,31E-04^a^ | 5,38E-05^a^ | 1,46E-04^a^ | 1,38E-04^a^ |
| hrs27 | 1884779 … 1884878 | 6,46E-05^a^ | 2,07E-04^a^ | 7,33E-05^a^ | 1,17E-04^a^ | 8,25E-05^a^ |
| hrs28 | 1892336 … 1892444 | 8,98E-05^a^ | 7,83E-05^a^ | 4,34E-05^a^ | 7,41E-05^a^ | 8,42E-05^a^ |
| hrs29 | 2354274 … 2354380 | 0^a^ | 1,57E-05^a^ | 4,47E-05^a^ | 1,18E-05^a^ | 0^a^ |
| hrs30 | 2455701 … 2455784 | 0^a^ | 1,87E-05^a^ | 0^a^ | 1,51E-05^a^ | 2,73E-05^a^ |
| hrs31 | 2519085 … 2519165 | 1,49E-04^a^ | 1,63E-03^a^ | 1,08E-03^a^ | 1,88E-03^a^ | 1,39E-03^a^ |
| hrs32 | 2611885 … 2611938 | 0^a^ | 0^a^ | 0^a^ | 0^a^ | 0^a^ |
| hrs33 | 914094 … 913974 | 2,96E-04^a^ | 4,01E-04^a^ | 4,06E-04^a^ | 5,91E-04^a^ | 2,63E-04^a^ |
| hrs34 | 415359 … 415287 | 0^a^ | 2,64E-05^a^ | 1,19E-05^a^ | 6,56E-06^a^ | 0^a^ |
| rprA | 1734418 … 1734308 | 0^a^ | 3,48E-04^a^ | 3,55E-04^a^ | 3,91E-04^a^ | 6,01E-04^a^ |
| **Non-coding RNA** | | | | | | |
| rmsB | 3465920 … 3467209 | 1,01E+00^a^ | 2,86E-01^b^ | 2,71E-01^b^ | 1,88E-01^b^ | 1,13E-01^b^ |
| **Genes** | | | | | | |
| *ppqA* | 3173704 … 3173784 | 6,38E-04^a^ | 2,15E-03^a^ | 6,98E-04^a^ | 2,94E-03^a^ | 9,42E-04^a^ |
| EAM_2938 | 569413 … 569255 | 1,04E-03^a^ | 1,62E-02^b^ | 7,47E-03^b^ | 1,53E-02^c^ | 3,29E-03^a^ |
| *orf18* | 594641 … 594606 | 0^a^ | 0^a^ | 0^a^ | 0^a^ | 0^a^ |

* The data that do not differ significantly from one another are marked with the same letter.
