# Supplementary material for: Comparative transcriptome analysis of a lowly virulent strain of Erwinia amylovora in shoots of two apple cultivars – susceptible and resistant to fire blight
Source: BMC Genomics. 2017 Nov 13;18:868. doi: 10.1186/s12864-017-4251-z (PMC5683332; doi:10.1186/s12864-017-4251-z)
Supplement: Supplementary file 9 — Genes of E. amylovora 650 coding for membrane proteins and differentially expressed in two apple cultivars – Idared and Free Redstar (I-24 h and FR-24 h) 24 h after inoculation. (DOCX 16 kb) [file 12864_2017_4251_MOESM9_ESM.docx]

| **Gene** | **Locus** | **Product** | **Fold change** | **FDR** | **COG/eggNOG** |
| --- | --- | --- | --- | --- | --- |
| Genes up-regulated in Idared (I-24h) | | | | | |
| *yghB* | EAMY_0467 | Protein DedA (Protein DSG-1) | 1.50 | 1.26E-03 | S |
| *yagU* | EAMY_0713 | Inner membrane protein YagU | 1.56 | 1.61E-03 | S |
| EAMY_1024 | EAMY_1024 | membrane protein | 1.62 | 1.26E-03 | S |
| *gltJ* | EAMY_1128 | putative glutamate aspartate ABC transportsystem, inner membrane component | 1.56 | 2.59E-02 | E |
| *yohC* | EAMY_1230 | Inner membrane protein YohC | 2.00 | 7.98E-08 | S |
| *ycfJ* | EAMY_1492 | Uncharacterized protein YcfJ | 1.56 | 7.85E-04 | M |
| *apbE* | EAMY_1563 | Thiamine biosynthesis lipoprotein apbEprecursor | 1.53 | 1.47E-02 | H |
| *lpp* | EAMY_1681 | Major outer membrane lipoprotein precursor | 1.76 | 1.31E-05 | S |
| *yeiH* | EAMY_2301 | putative PSE family transporter | 2.07 | 3.58E-03 | S |
| EAMY_2393 | EAMY_2393 | putative ABC transport system, inner membranecomponent | 1.66 | 1.74E-02 | P |
| *lrgA* | EAMY_3280 | UPF0299 membrane protein PM0880 | 1.88 | 1.05E-02 | S |
| Genes up-regulated in Free-Redstar (FR-24h) | | | | | |
| *aaeB* | EAMY_0293 | p-hydroxybenzoic acid efflux pump subunit aaeB | 1.64 | 2.81E-03 | D |
| *ompU* | EAMY_0413 | outer membrane protein | 2.77 | 1.01E-11 | S |
| *acrA* | EAMY_1009 | Membrane-fusion protein | 1.70 | 1.02E-06 | V |
| *yncD* | EAMY_1080 | probable tonB-dependent receptor yncD precursor | 1.56 | 1.02E-03 | P |
| *yeiU* | EAMY_2308 | Inner membrane protein yeiU | 1.53 | 1.57E-03 | I |
| *emrA* | EAMY_2718 | Multidrug resistance protein A | 2.06 | 1.05E-09 | V |
| *yqjF* | EAMY_3107 | Inner membrane protein yqjF | 1.72 | 4.12E-02 | S |
| *yhhQ* | EAMY_3502 | Inner membrane protein yhhQ | 1.59 | 9.78E-03 | S |
| *prtF* | EAMY_3577 | Type I secretion system, TolC-family protein | 2.51 | 4.49E-03 | M |
| *prtE* | EAMY_3578 | Type I secretion system, membrane-fusionprotein | 1.78 | 1.92E-03 | U |
| *yebN* | EAMY_3586 | putative YebN family transporter | 1.85 | 3.93E-04 | S |
| *yhjW* | EAMY_3617 | UPF0141 inner membrane protein yhjW | 1.64 | 9.71E-05 | M |
| *yidC* | EAMY_3682 | Inner membrane protein yidC | 1.54 | 1.74E-04 | U |

Table S8. Genes of *E. amylovora* 650 coding for membrane proteins and differentially expressed in two apple cultivars – Idared (I-24h) and Free Redstar (FR-24h) 24h after inoculation.
