# Supplementary material for: Comparative transcriptome analysis of a lowly virulent strain of Erwinia amylovora in shoots of two apple cultivars – susceptible and resistant to fire blight
Source: BMC Genomics. 2017 Nov 13;18:868. doi: 10.1186/s12864-017-4251-z (PMC5683332; doi:10.1186/s12864-017-4251-z)
Supplement: Supplementary file 12 — The content of different COG/eggnog categories among genes of different expression between two time points after inoculation–24 h and 6 days. UP –up-regulated, DOWN –down-regulated, I –Idared, FR –Free Redstar, 6d −6 days. (PDF 122 kb) [file 12864_2017_4251_MOESM12_ESM.pdf]

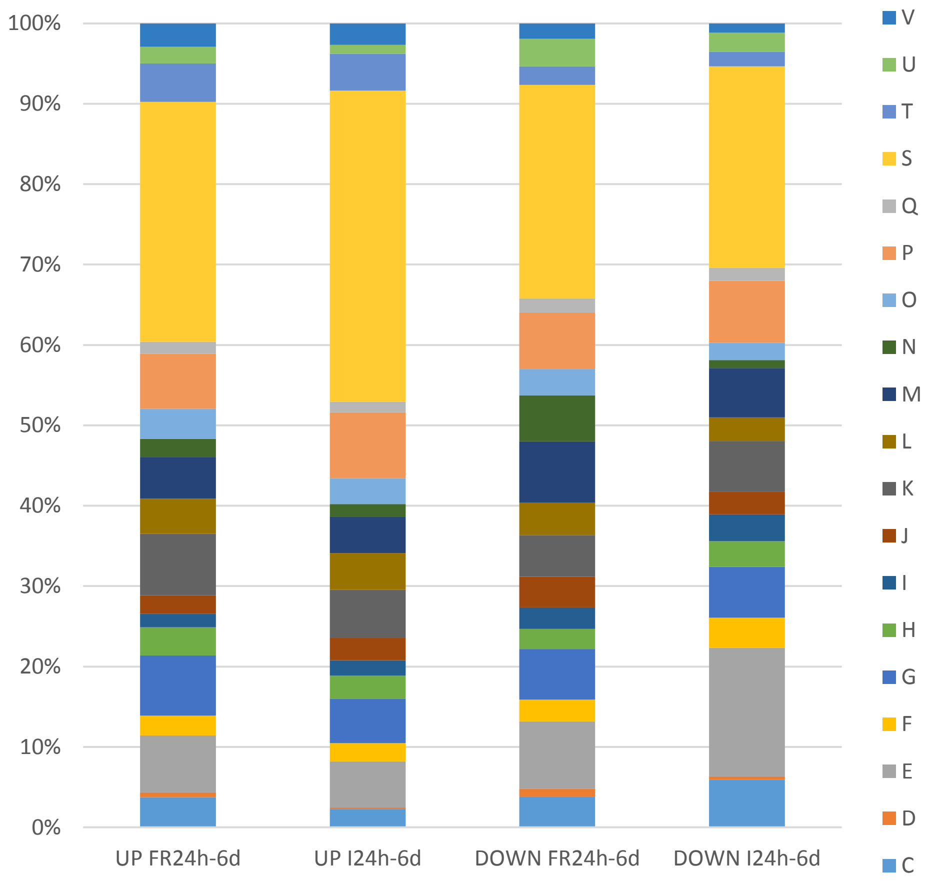

Fig. S2. The content of different COG/eggNOG categories among genes of different expression between two time points after inoculation – 24 h and 6 days. UP – up-regulated, DOWN – down-regulated, I – Idared, FR – Free Redstar, 6d – 6 days.
