# Supplementary material for: Comparative transcriptome analysis of a lowly virulent strain of Erwinia amylovora in shoots of two apple cultivars – susceptible and resistant to fire blight
Source: BMC Genomics. 2017 Nov 13;18:868. doi: 10.1186/s12864-017-4251-z (PMC5683332; doi:10.1186/s12864-017-4251-z)
Supplement: Supplementary file 13 — Change of expression of known genes involved in pathogenicityof Erwinia amylovorabetween24 h (FR-24 h) and 6 days (FR-6d) after inoculation on Idared and on Free Redstar (PDF 322 kb) [file 12864_2017_4251_MOESM13_ESM.pdf]

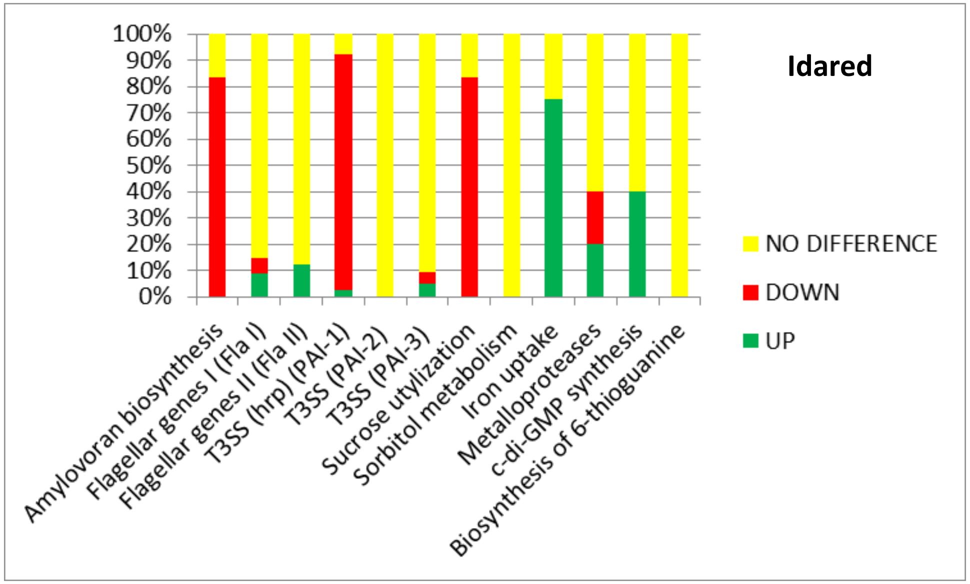

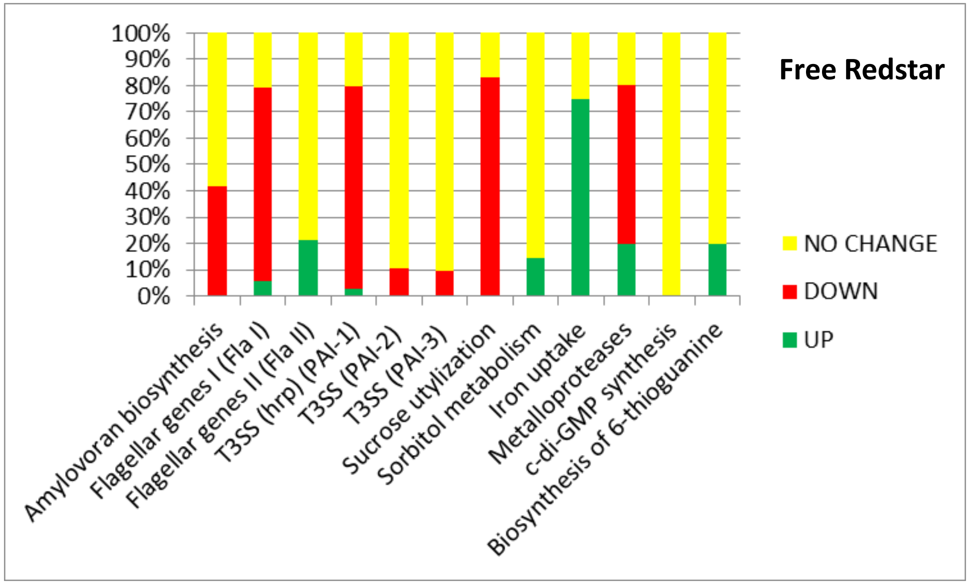

Fig. S3.
Change of expression of known genes involved in pathogenicity of *Erwinia amylovora* between 24 h (FR-24h) and 6 days (FR-6d) after inoculation on Idared and on Free Redstar
