# Supplementary material for: Comparative transcriptome analysis of a lowly virulent strain of Erwinia amylovora in shoots of two apple cultivars – susceptible and resistant to fire blight
Source: BMC Genomics. 2017 Nov 13;18:868. doi: 10.1186/s12864-017-4251-z (PMC5683332; doi:10.1186/s12864-017-4251-z)
Supplement: Supplementary file 16 — Primers used for qRT-PCR validation of RNAseq data. (DOCX 14 kb) [file 12864_2017_4251_MOESM16_ESM.docx]

Table S13. Primers used for qRT-PCR validation of RNAseq data

| Gene Id | Name/Putative function | Forward primer 5’-3’ | Reverse primer 5’-3’ | Amplicon bp |
| --- | --- | --- | --- | --- |
| *amsB* | Amylovoran biosynthesis glycosyl transferase AmsB/ polypeptide N-acetylgalactosaminyltransferase activity | GCGGTAATTTATAGGCTTTGTAGG | AAGTATTCTCTGTTCTGGCTG GAC | 85bp |
| *cho* | Putative excinuclease cho/ hydrolase activity | GATTTTTGCGCAGTCGTTTATTG | ACTGCCGATGAAGCCCGTATGTGG | 142 |
| *clpX* | ATP-dependent Clp protease ATP-binding subunit clpX/ Molecular functioni-ATP binding, peptidase activity, zinc ion/ biological process protein folding (chaperone activity) | TGACCGATAAACGCAAAGACG | TCCACGCATTCATCGCAGACATA | 124 |
| *cysD* | 3'-phosphoadenosine 5'-phosphosulfate sulfotransferase (PAPS reductase)/FAD synthetase and related enzymes"/ adenylylsulfate kinase activity, ATP binding | CGTCTTCCCGCTGTCTAACTG | CGGTCGTCATCAATCATCATCAAC | 144 |
| *dfoA* | L-lysine 6-monooxygenase involved in desferrioxamine biosynthesis /(NADPH) activity | GCGAATATCTGCTCAACAAAG | TCCGTCAGCAGATCGTAATAG | 103 |
| EAMY 0934 | Putative lipoprotein/ transferase activity | CATCGTGGAACAGGCTAAGG | ATCGGGACGGCTGGTATCTG | 93 |
| *fliC1* | Flagellin/ molecular function-structural molecule activity/ biological process- bacterial-type flagellum-dependent cell motility | TGAGCGGAAGACAGGTTAGTGGTG | CGCTGGCTCTGCTGGACAAAG | 130 |
| *hrpN* | Harpin HrpN/ Biological Process -modulation by symbiont of host defense-related programmed cell death/ elicits HR in non-hosts and is also required for pathogenicity in host plants. | CCTGAGCGGGCCGGTGGACTAC | TCGCCCGATCGCCTTTATTGAC | 146 |
| *oppC3* | Putative ABC transport system, inner membrane component/  Biological Process-transport | GCGGAAGATGAGCCACGGGTTGA | ATTTCGCTGCTGCTGTGGGTAGAG | 123 |
| *paaK* | Coenzyme F390 synthetase II/ phenylacetate-CoA ligase activity | CTGTCCGCG TAATGAGGTC | ACGTGTCGCCGGTTGAGTG | 140 |
| *xseA* | Exodeoxyribonuclease 7 large subunit/ Molecular function-exodeoxyribonuclease VII activity, nucleic acid binding/ Biological process-DNA catabolic process | TTGAGCAGCGCCATAAACAGC | CCGGCAGGGAAGGGTCACG | 126 |
